# BCAT1 expression associates with ovarian cancer progression: possible implications in altered disease metabolism

**DOI:** 10.18632/oncotarget.5159

**Published:** 2015-09-10

**Authors:** Zhi-Qiang Wang, Adnen Faddaoui, Magdalena Bachvarova, Marie Plante, Jean Gregoire, Marie-Claude Renaud, Alexandra Sebastianelli, Chantal Guillemette, Stéphane Gobeil, Elizabeth Macdonald, Barbara Vanderhyden, Dimcho Bachvarov

**Affiliations:** ^1^ Department of Molecular Medicine, Laval University, Québec PQ, Canada; ^2^ Centre de recherche du CHU de Québec, L'Hôtel-Dieu de Québec, Québec PQ, Canada; ^3^ Department of Obstetrics and Gynecology, Laval University, Québec PQ, Canada; ^4^ Centre de recherche du CHU de Québec, CHUL, Québec PQ, Canada; ^5^ Faculty of Pharmacy, Laval University, Québec PQ, Canada; ^6^ Department of Cellular and Molecular Medicine, University of Ottawa, Ottawa, ON, Canada

**Keywords:** BCAT1, ovarian cancer, cancer metabolism, metastasis, DNA hypomethylation

## Abstract

Previously, we have identified the branched chain amino-acid transaminase 1 (*BCAT1*) gene as notably hypomethylated in low-malignant potential (LMP) and high-grade (HG) serous epithelial ovarian tumors, compared to normal ovarian tissues. Here we show that *BCAT1* is strongly overexpressed in both LMP and HG serous epithelial ovarian tumors, which probably correlates with its hypomethylated status. Knockdown of the *BCAT1* expression in epithelial ovarian cancer (EOC) cells led to sharp decrease of cell proliferation, migration and invasion and inhibited cell cycle progression. *BCAT1* silencing was associated with the suppression of numerous genes and pathways known previously to be implicated in ovarian tumorigenesis, and the induction of some tumor suppressor genes (TSGs). Moreover, *BCAT1* suppression resulted in downregulation of numerous genes implicated in lipid production and protein synthesis, suggesting its important role in controlling EOC metabolism. Further metabolomic analyses were indicative for significant depletion of most amino acids and different phospho- and sphingolipids following *BCAT1* knockdown. Finally, *BCAT1* suppression led to significantly prolonged survival time in xenograft model of advanced peritoneal EOC. Taken together, our findings provide new insights about the functional role of *BCAT1* in ovarian carcinogenesis and identify this transaminase as a novel EOC biomarker and putative EOC therapeutic target.

## INTRODUCTION

Epithelial ovarian cancer (EOC) accounts for 4% of all cancers in women and is the leading cause of death from gynecologic malignancies [1]. Despite treatment improvements, long-term survival rates for patients with advanced disease remain disappointing [2]. The molecular basis of EOC initiation and progression is still poorly understood. To establish novel therapeutic and diagnostic strategies against this deadly disease, it is essential to understand its molecular pathology.

Recently, the importance of epigenetic perturbation of gene regulation in cancer [3], including EOC [4], has begun to be more fully appreciated. The most studied epigenetic alteration is DNA methylation, the addition of a methyl moiety to the cytosine-5 position within the context of a CpG dinucleotide, mediated by DNA methyltransferases [3]. Similar to other malignancies, aberrant DNA methylation occurs in EOC and contributes to ovarian tumorigenesis and mechanisms of chemoresistance [4]. Applying a more global array-based technology, several studies have demonstrated that DNA methylation changes in ovarian cancer are cumulative with disease progression and chemotherapy (CT) resistance [5–7]. Using a similar approach (methylated DNA immunoprecipitation coupled to CpG island tiling arrays) we have recently shown that DNA hypermethylation occurs in all (including less invasive/early) stages of ovarian tumorigenesis. Interestingly, advanced EOC was exclusively associated with DNA hypomethylation of a number of oncogenes, implicated in cancer progression, invasion/metastasis and probably chemoresistance [8]. The cytosolic form of the branched chain amino-acid transaminase 1 (*BCAT1*) was among the genes identified to be notably hypomethylated in low-malignant potential (LMP) and high grade (HG) serous EOC tumors [8]. *BCAT* enzymes comprise two isoforms: *BCAT1* (cytosolic) and BCAT2 (mitochondrial). Both isoforms regulate the first step in degradation of branched-chain amino acids (BCAAs) including leucine, isoleucine and valine, which are essential for cellular metabolism and growth [9]. While *BCAT2* is expressed in most tissues, *BCAT1* expression is rather restricted to some highly specialized tissues, including brain, ovary and placenta [9]. Several groups have confirmed that *BCAT1* is involved in cell proliferation, cell cycle progression, differentiation and apoptosis [10, 11]. Recent studies were indicative for the important role of *BCAT1* in the progression of several malignancies, including medulloblastomas, nonseminomas, colorectal and nasopharyngeal carcinomas and glioblastomas [12–16]. Moreover, the role of BCAAs metabolism in cancer pathogenesis has been also a topic of interest [17].

This prompted us to further investigate if *BCAT1* displays elevated expression levels in serous EOC tumors with different malignant potential, and whether this gene is functionally implicated in EOC dissemination. Here we present experimental data indicative for strong *BCAT1* overexpression in LMP and HG serous EOC tumors, which probably correlates with its hypomethylated status. We also show that blocking *BCAT1* expression inhibits proliferation, migration, invasion and peritoneal dissemination, possibly through altering EOC metabolism.

## RESULTS

### Overexpression of *BCAT1* in both LMP and HG serous EOC tumors

Previously, we have identified the *BCAT1* gene as hypomethylated in LMP and HG EOC tumors, when compared to normal tissues [8]. Here, we further evaluated *BCAT1* protein expression by immunohistochemistry (IHC) in serous EOC tumors and ovarian normal tissue samples, using tissue microarrays (TMAs). Our TMAs included triplicate cores of 117 serous EOC tumors, including 13 LMP tumors and 104 HG ovarian tumors. Thirteen normal ovarian tissue samples were also included as controls. Table 1 shows the major clinical characteristics of these patients for whom extensive follow-up clinical data (up to 5-years) were available. The age ranged from 41 to 83 years (median: 66 years). High-grade tumors were all grade 3 (100%) including stage III (69%) and stage IV (31%) tumors. The majority of patients (93%) received a combination of platinum and paclitaxel. The median baseline CA125 was around 800. Forty percent of the patients had a progression or a recurrence within the first 6 months of follow-up; for 39% of the patients the progression-free survival (PFS) interval was in the range of 7 to 24 months, and 21% of the patients displayed PFS values higher than 25 months (Table 1).

**Table 1:** Detailed patients’ clinicopathological characteristics

Variable	Range	n/total	%
Age (years)			
	≥ 65	64/130	49
	< 65	66/130	51
Median	66		
Tissue/tumor type			
	Normal	13/130	10
	LMP	13/130	10
	High-grade	52/130	40
	OM	52/130	40
Grade			
	3	104/104	100
Stage			
	III	72/104	69
	IV	32/104	31
Chemotherapy^1^			
	platinum+taxol	97/104	93
	Other	13/104	7
CA125			
	≥ 800	47/104	45
	< 800	53/104	55
PFS (months)^2^			
	0–6	41/103	40
	7–24	40/103	39
	> 25	22/103	21

1 All patients were subjected to adjuvant therapy.

2 Extended follow-up, including PFS values, were available for 103 patients.

OM - omental metastasis

**Figure 1:**
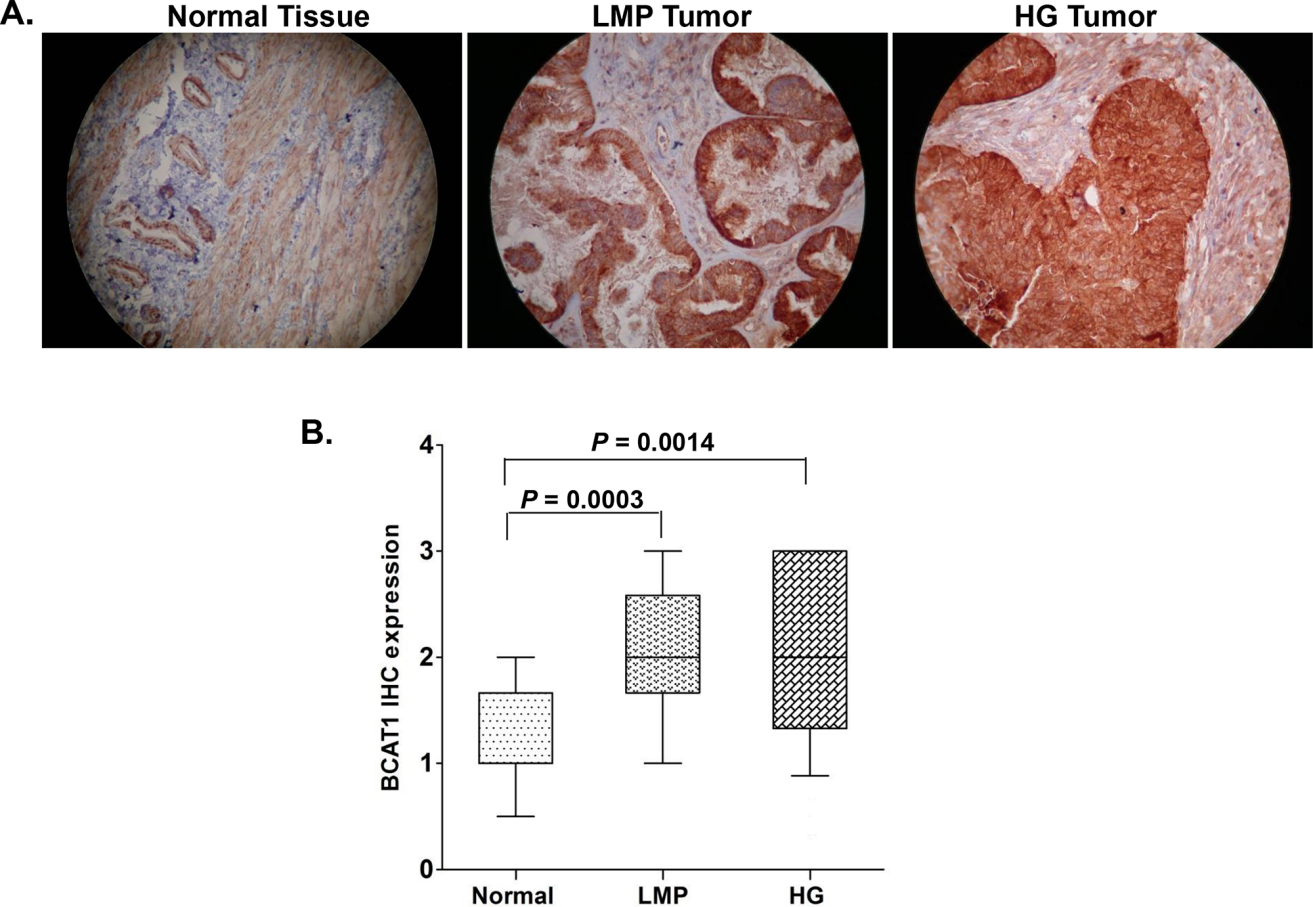
Analysis of *BCAT1* expression in serous EOC tumors by IHC **A.** Representative IHC images of *BCAT1* protein expression in normal ovarian tissues, low-malignant potential (LMP) tumors and high-grade (HG) tumors. **B.** Box-plot presentation of *BCAT1* protein expression levels in normal ovarian tissues, LMP tumors and HG tumors.

As seen from Figure 1, *BCAT1* displayed significantly higher expression in LMP tumors and HG serous EOC tumors, when compared to normal tissues (*p* = 0.0003 and *p* = 0.0014, respectively), which correlates with *BCAT1* hypomethylation status in these tumor types. However, we did not observe any significant differences between the levels of *BCAT1* expression and patients’ PFS values (*p* = 0.0901; see Supplemental Figure 1), which suggests that staining intensity for *BCAT1* in pre-treatment surgical specimens is not predictive of PFS.

### Phenotype analysis of *BCAT1* suppression in EOC cells: possible implications in EOC cell proliferation, cell cycle control, migration and invasion

Next, we decided to verify if short-hairpin RNA (shRNA)-mediated *BCAT1* gene knockdown could produce any cancer-related phenotypic changes in EOC cells. We tested several EOC cell lines for endogenous *BCAT1* protein expression by Western blot analysis (see Supplemental Figure 2). Among these, the SKOV3 and the A2780s cell lines displayed strong *BCAT1* expression and were further used to generate stably transfected shRNA-*BCAT1* clones. Clone selection for further analyses was based on semi-quantitative (sq) RT-PCR and Western blot validation of the *BCAT1* mRNA/protein expression in selected clones, compared to empty vector-transfected clones. Among the clones analyzed, the SKOV3 shRNA-*BCAT1* knockdown clones sh-B1 and sh-B2 and the A2780s clones sh-A1 and sh-A2 displayed significant decrease of *BCAT1* expression levels compared to the mock-transfected control (Supplemental Figure 2) and were selected for further analyses.

**Figure 2:**
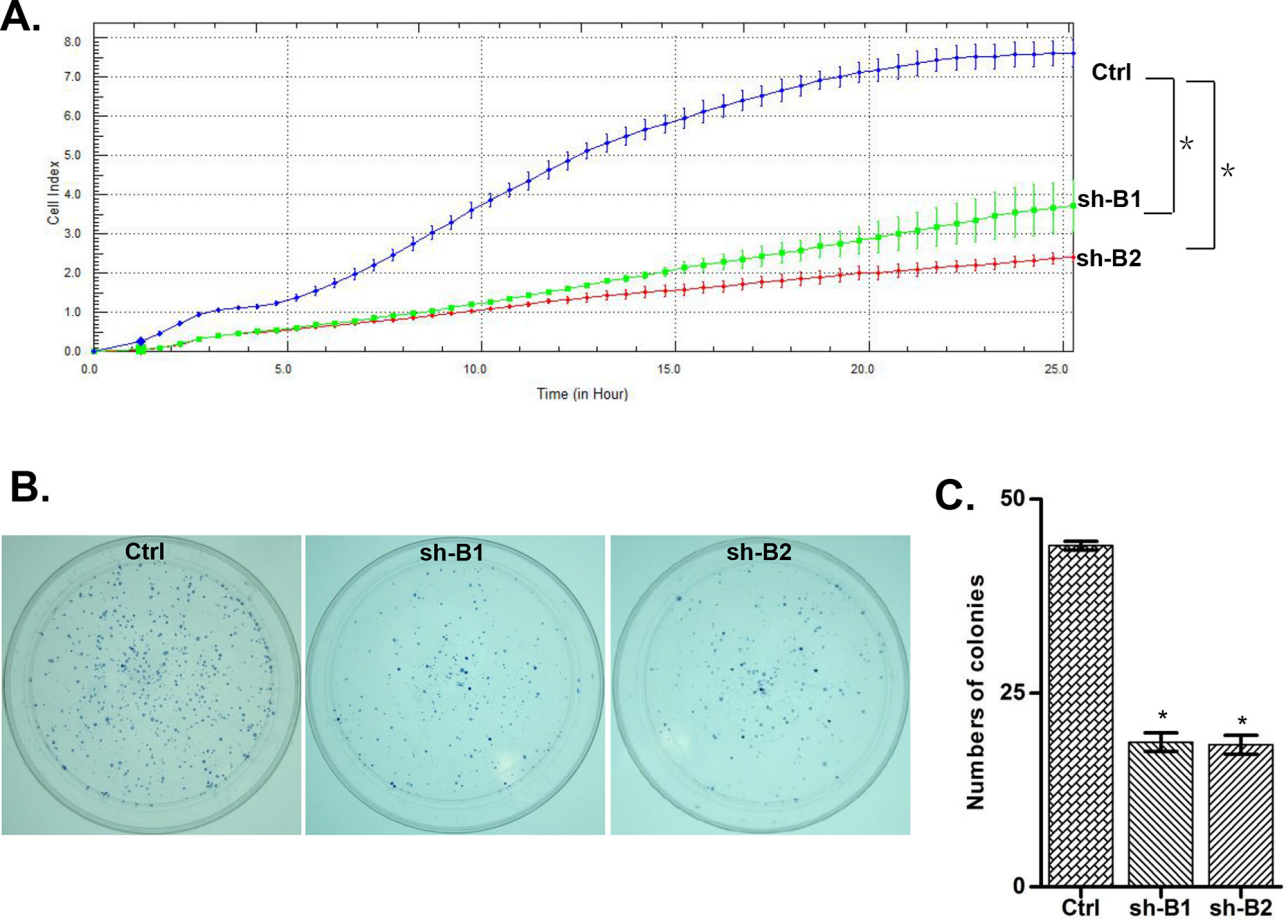
ShRNA-directed knockdown of the *BCAT1* expression in SKOV3 cells **A.** effect of BCAT1 knockdown clones 1 and 2 (sh-B1 and sh-B2) on cell proliferation, compared to the control clone (Ctrl); **B.** Representative images of colony formation assays following BCAT1 knockdown; **C.** Quantitative determinations (graph-bars) of data obtained: results are expressed as numbers of sh-B1- and sh-B2-induced colony formation compared to the Ctrl-induced colony formation numbers. Differences were determined using the Student's *t*-test. Error bars denote mean ± SEM; *indicates statistical significance (*p* < 0.05)

**Figure 3:**
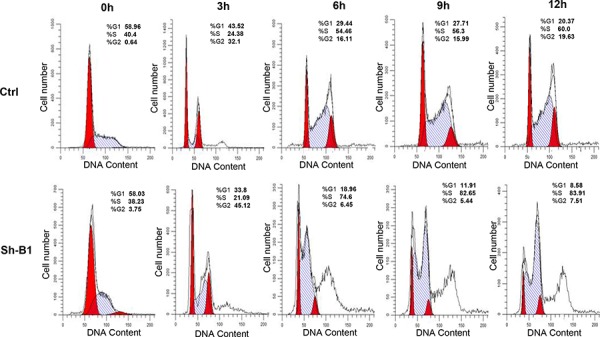
Effect of *BCAT1* suppression on cell cycle control in SKOV3 cells Cell-cycle profile was examined by flow cytometry and percentages of cells in G0/G1, S, and G2/M phase in the shRNA-BCAT1 clone 1 (sh-B1) were compared to the mock-transfected control (Ctrl) clone. Propidium iodide staining shows an increased fraction of cells in the S-phase and corresponding decrease of cells in both G1- and G2/M-phase at 9 and 12 h after removing hydroxyurea in the shRNA-*BCAT1* clone B1 (sh-B1), when compared with the control clone (Ctrl).

**Figure 4:**
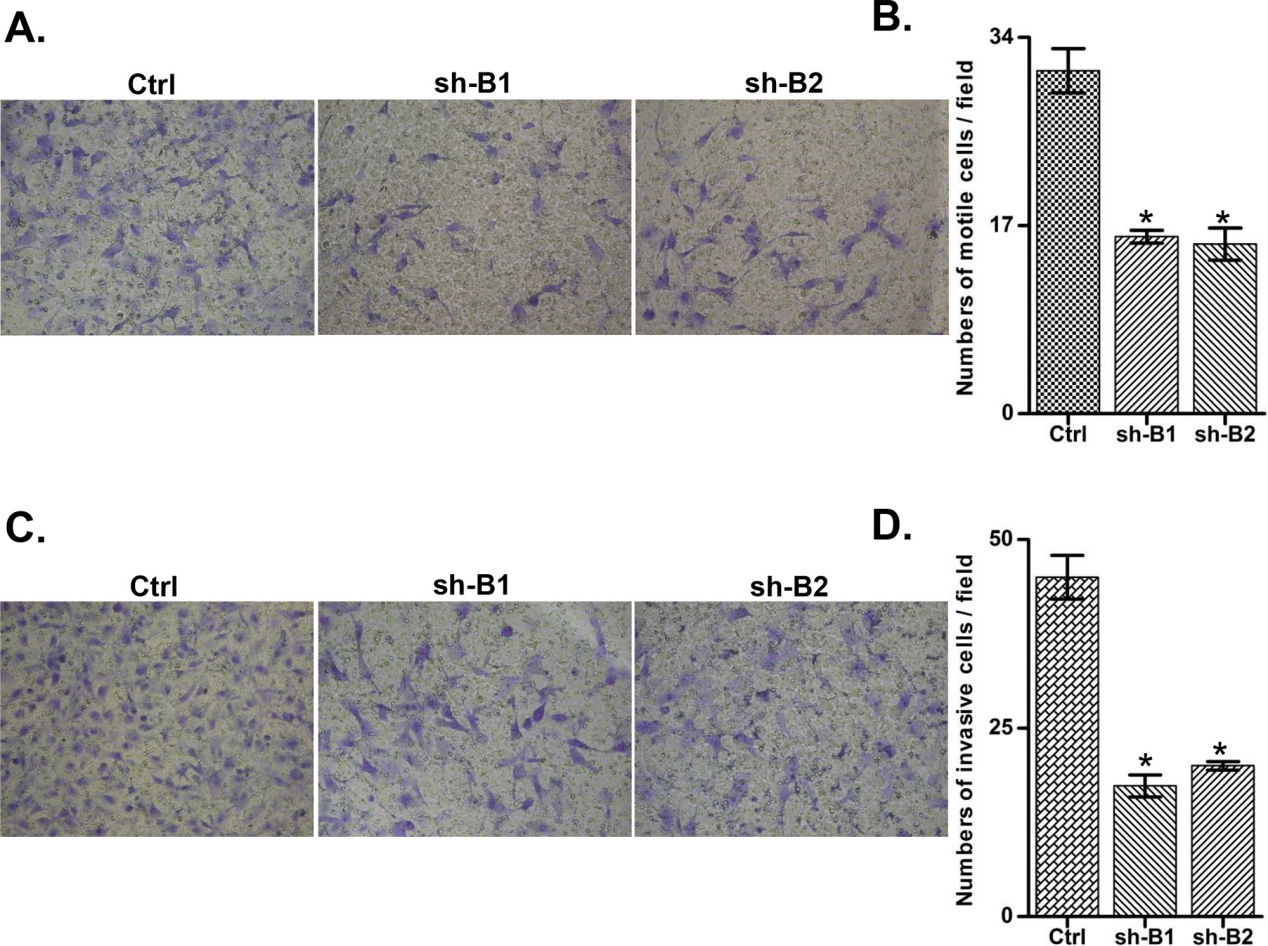
Effect of *BCAT1* knockdown on SKOV3 cell migration and invasion **A.** Migration was assessed using Boyden-chamber assay. Cells from the shRNA-*BCAT1* clones 1 and 2 (sh-B1 and sh-B2) and the control (Ctrl) clone were seeded into the upper chambers in 0.1% FBS containing medium at a density of 2.5 × 10^4^ per well, and 600 μl of 1% FBS containing medium was placed in the lower chamber as a chemoattractant. After 24 h at 37°C in 5% CO2, the cells were fixed with cold methanol and stained with blue trypan solution. Migrated cells on the underside of the filter were photographed and counted by phase contrast microscopy. **C.** Cell invasion was assayed in a similar way, as the upper chambers were coated with Matrigel. Here, NIH3T3 conditioned medium was added in the lower chamber as a chemoattractant (see Materials and Methods for details). All experiments were performed in triplicate. For each experiment, cell number was calculated as the total count from 10 random fields per filter (at magnification × 40). The bar graphs in panels **B.** and **D.** represent quantitative determinations of data obtained by selecting 10 random fields per filter under phase contrast microscopy and results are expressed as % change of the sh-G1 and sh-G2 clones over the Ctrl clone. Differences between shRNA-*BCAT1*-transfected and vehicle-transfected SKOV3 cells were determined by a Student's *t*-test; error bars denote mean ± SEM; *indicates statistical significance (*p* < 0.05).

We investigated the impact of the *BCAT1* gene suppression on SKOV3 cell proliferation, cell cycle control, migration, invasion, and sensitivity to cisplatin and paclitaxel (drugs, conventionally used for first-line EOC therapy). The *BCAT1* gene knockdown led to sharp decrease of the number of viable adherent cells (represented by cell index), compared to control cells (Figure 2A). This observation was further supported by the reduced number of colony formation upon *BCAT1* suppression (Figure 2B and 2C). Moreover, *BCAT1* depletion induced S cell cycle arrest which could explain the drastic reduction in the proliferation rates of these EOC cells observed earlier (Figure 3). Additionally, *BCAT1* suppression significantly inhibited both migration and invasion of SKOV3 cells. Indeed and as shown in Figures 4A and 4B (migration) and Figures 4C and 4D (invasion), the numbers of SKOV3 cells that passed through the filter using the sh-B1 and sh-B2 clones were remarkably less than in the negative control (Ctrl) clone.

Quite similar results were obtained when performing functional analyses in A2780s cells following *BCAT1* knockdown (see Supplemental Figure 3).

Finally, *BCAT1* knockdown had no significant impact on SKOV3 cisplatin and paclitaxel sensitivity (see Supplemental Figure 4).

### Molecular mechanisms of *BCAT1* action in EOC cells

To better understand the molecular mechanisms of *BCAT1* action in EOC cells, we compared the gene expression of the previously selected shRNA-*BCAT1* SKOV3 clones (sh-B1 and sh-B2) against the corresponding control clone (Ctrl). All microarray experiments were performed in duplicates, as two hybridizations were carried out for each of the two *BCAT1* knockdown cell clones against the corresponding control, using a fluorescent dye reversal (dye-swap) technique. For both comparisons, a subset of common differentially expressed genes was selected by initial filtering on confidence at *p*-value ≤0.05, followed by filtering on expression level (≥2 fold). Using these stringent selection criteria, we found 878 genes were upregulated and 979 genes were downregulated in SKOV3 cells upon *BCAT1* knockdown; among these, the *BCAT1* gene displayed significant medium suppression value (−8.34 fold; see Supplemental Table 1B). Table 2 shows a list of selected functionally related groups of genes that were differentially expressed (≥2.0-fold at *p* value ≤0.05) in SKOV3 cells following *BCAT1* suppression. As seen from Table 2, genes with previously shown implication in mechanisms of metabolism, signal transduction, regulation of transcription, transport, cell growth & proliferation and cell cycle, were predominantly or exclusively suppressed, while *BCAT1* knockdown was related with the induction of some genes related with immune & inflammatory response and cell adhesion. Supplemental Table 1 shows the complete list of the differentially expressed genes (≥2.0-fold at *p* value ≤0.05) following *BCAT1* knockdown in SKOV3 cells.

**Table 2:** Selected differentially expressed gene groups in SKOV3 cells upon *BCAT1* knock-down

	**A. Upregulated genes**
metabolism	*ZNF404, ZNF556, ZNF608, ZNF626, ZNF654, ZNF781, AGBL5, CTSV, CTSZ, MIPEP, ZDHHC3, ADAMTS1, ADAMTS5, ADAMTS6, MMP13, PLAU, PROC, PRSS23, SENP2, C3orf38, USP46, LMLN, MME, RPL14, RPL15, RPL21, RPL24, TRIM6, IFT172, SPINT2, TCEAL5, TCEAL8, INSIG1, INSIG2, RBM3, NAP1L1, NAP1L2, RPL22, RPL27A, LACTB, DZIP1L, FSTL1, USPL1, ZC3H13, HNRNPA3, HNRNPH3, JRK, KLF8, LSM3, MID1, CXXC5, PHF11, SACS*
regulation of transcription	*CDKN2D, FAM172A, LMCD1, LPXN, PEX14, SERTAD2, ANKRD10, ARID5B, CTNNB1, ELL2, ETS1, FOXP1, HDAC6, HMGB1, HSBP1, JUN, KDM3A, KLF6, KLF7, MED4, MEIS1, MNT, MPHOSPH8, MSL3, MYCBP, NFKBIZ, NFYA, PFDN5, POU5F1, PPP1R13L, RBPJ, RUNX1T1, RUNX2, SAP18, SAP30BP, SMARCA4, SNAI1, SNAI2, STAT1, TAF9B, TARDBP, TBX3, TCEB2, TCF4, TSHZ3, WWTR1, EIF4H, EIF5, EIF5A2, MRPL43, RPSA, PAIP2B*
signal transduction	*PTPRB, PTPRS, SIRPA, PDCL, PDE11A, PDE4A, PDE4DIP, PLCB4, ARL4C, CETN2, GNAI2, GNB4, MRAS, RAB9B, RAP2A, RAP2B, CSF2, CXCL12, EDN1, IL11, TNFSF12, WNT5A, GPR126, GPRC5A, S1PR1, AKT3, CAMKK1, COL4A3BP, DYRK2, IP6K1, IP6K2, JAK1, MAP2K3, MYLK, NRBP2, PIK3CB, PRKAB2, PRKCA, SIK1, TRIO, CDK6, CLK1, NDRG1, NUCKS1, ACVR2B, EFNA5, EPHA4, THRB, ARHGAP1, ARHGAP23, GFRA1, JMJD6, LRPAP1, TNFRSF19*
transport	*PLIN2, VAMP8, PLIN3, STXBP6, CYB5B, CYBRD1, KCTD10, ANO7, CATSPER1, GABRQ, TRPM2, AP2M1, COG6, COPZ2, GGA2, PCTP, S100A6, SEC13, SEC22A, SLC9A9, STEAP3, SYT15, TMED9, UCP2, UCP3, APOL1, ATP13A3, ECM1, RBP4, TINAGL1, KPNA4, NUTF2, APOL3/APOL4, FAM101A/ZNF664-FAM101A, MFSD1, MFSD9, S100A3, SNX12, ABCA1, ABCC4, ABCC5, ATP2B4, ATP8A2, ITGB1BP1, PDZK1, SLC16A3, SLC2A1, SLCO2B1, TM9SF2*
immune & inflammatory response	*PSG1, PSG11, PSG3, PSG4, PSG6, PSG7, PSG9, IL7R, SRGN, PSG8, IGFN1, ZC3HAV1*
cell growth	*ACTC1, LOXL1, LOXL2, FGF5, GAS6, GDF6, INHBA, PTN, TGFB2, NRG1, FN1, KRT7, KRT8*
cell adhesion	*MEGF8, CDH13, CDH15, CDH2, CDH6, NKTR, PCDH20, MCAM, ACTC1, ACVR2B, CTNNB1, LMO7, MRAS, MYH10, MYL6, PTEN, TCF4*
	**B. Downregulated genes**
metabolism	*A4GALT, AADAT, AARS, ACAA2, ACOT1, ACOT2, ACOX2, AGPAT9, AHCYL1, AKR1B10, AKR1C1/AKR1C2, AKR1C3, ALDH3A1, ALDH3A2, ALDH9A1, ALOX5, AMACR, ASNS, ASPH, ASRGL1, BCAT1, CA2, CARS, CBR4, CBS, CES1, CHST6, COX5A, CYP2J2, DGAT2, DHX58, DIMT1, EBP, ECI1, EGLN3, EPRS, FA2H, FUT8, GALNT12, GALNT14, GARS, GATM, GCLC, GDA, GMPR2, GNPNAT1, GOT1, GPD1L, GPT2, GPX1, GSR, GSTT2/GSTT2B, HGD, HIBADH, HS3ST1, IARS, IDH1, IDH2, ISYNA1, KYNU, LRAT, MAN2A2, MARS, MGAT3, MPST, MTHFD2, MTHFD2L, MTHFS, NAPRT1, NCF2, NDUFC1, NDUFS1, NEDD4, NMNAT2, NMNAT3, PAPSS2, PDE8B, PGD, PHGDH, PIGF, PLCD3, PLCG2, PNPLA4, POLRMT, PPCDC, PSAT1, PTGS1, PTGS2, PTRH2, PYGB, RAB27B, RASD1, RHOU, RNF135, RPL4, SARS, SEPSECS, SHMT2, SMOX, SOD2, SORD, SQRDL, SRD5A3, SRXN1, ST3GAL1, ST6GAL1, SULT1A1, SULT1A2, SULT1A3/SULT1A4, TDO2, TGM2, TMOD1, TMX3, TRIM2, TST, UBB, UBC, UGT2B10, UGT2B7, UQCRC2, WARS, YARS, ZNRF1, DDHD1, ENPP5, LOXL4, PLA2G7, SDF2, USP53, VNN3, ARL4D, DDX52, ELAC1, FBXO25, NEDD8, PNP, RBBP8, RDH10, TARS, TOP2A, TRIM69, UGDH, UHRF2, ABHD3, CTAGE5, DDAH2, DHRS13, DHRS7, DHRSX, FAAH2, GLT8D2, GNPDA2, GPX8, HECTD1, VNN2, DLL1, GBP5, GCNT3, HS6ST2, NLGN4X, PDE6A, PIGP, SULF2, BLMH, CLGN, CPE, LONP1, MEST, PSMA3, PSMA6, PSME2, SENP6, UCHL1, USP18, USP3, C1R, C1S, CPD, PCSK5, PCSK6, PRSS2, PSMC6, CELA3B, CPVL, ADAM23, ANPEP, CPM, ECEL1, SPPL2A, TMPRSS3, GRSF1, JAKMIP1, MRPL46, RPL19, RPL23A, RPS29, SLPI, AHRR, CDC37L1, MKKS, MRPS35, MRPS36, UGT2B28, FGG, LGALS3, SERPINA3, SERPINB5, VWF, HIST1H1A, KLF14, RBPMS2, TRIM31, GRPEL2, CPEB4, ZBED3, FBXO33, LARP6*
signal transduction	*IBSP, ICAM2, SPRY4, ADAP2, ARHGEF2, NGEF, CLEC2B, CNIH1, FLOT2, PDLIM4, CISH, EFHD1, ERRFI1, RALGAPA1, SH3BGR, SHC2, TMEFF2, TMEM33, TMTC2, AIM1, NMB, NMU, SMOC2, IFRD1, RADIL, RASEF, ANXA1, PECAM1, SNTB1, DAAM1, GKAP1, GRB10, JKAMP, RGS16, S100P, IGFBP2, IGFBP5, MNAT1, NKD2, CGRRF1, GAREM, SAMD13, SAMD5, CCL26, IL18, IL1A, IL7, SCG2, SPP1, TSLP, VAV3, ANXA3, BIRC3, RAB39B, CCR7, F2RL2, FZD1, FZD8, GPR135, GPR64, LGR6, LPHN3, OR7E24, RXFP1, TAPT1, CAMK2D, DAPK2, ITPK1, ITPKA, MAP2K1, MAP2K5, MAP2K6, MAP3K5, MAPK6, MARK1, NME3, PCK2, PFKM, PIK3R1, PRKCH, PXK, SGK2, ULK1, NEK1, SRP72, STK39, TRIB3, HKDC1, MAST4, NME5, STK32A, BMPR1B, DDR2, ERBB2, PDGFRL, RIPK2, AR, ESR1, NR0B1, NR1D1, PGR, PPARG, RARA, ARHGAP24, ARHGAP25, ARHGAP26, PEX12, TMC5, TMEM128, TMEM170A, TMEM199, TMEM87A, ACP6, DUSP6, EYA4, INPP4B, NT5C3A, PPM1A, PPP1R1A, PPP1R1B, PSPH, PTP4A2, SGPP2, PTPDC1, DUSP5, EYA2, PPM1E, UBLCP1, DUSP14, ALPP, KTN1, CHRNA3, CLDN3, EPHA10, HLA-B, HLA-E, HLA-F, IL18R1, IL1R2, IL6ST, ITGA6, ITGB2, MSR1, PLXNC1, SFRP1, THBD, TLR3, TNFRSF11B, UNC5B*
regulation of transcription	*ANKRA2, CRLF3, GAS7, AJUBA, ATF3, ATF4, BATF3, CEBPA, CEBPB, CEBPG, DDIT3, DTX1, E2F5, EHF, ETV4, FOS, FOXA1, FOXC1, FOXP2, FOXQ1, GATA5, GATA6, GLI1, GSC, GTF2A2, HDAC4, HEXIM1, HEY1, HEYL, HIF1A, HLF, HOXC9, HOXD1, HOXD8, ID1, ILF2, IRF2BP2, JDP2, JMY, KCTD1, KLF4, KLF5, MECOM, MEF2A, MESP1, MSX1, MXD1, NAB1, NCOA7, NEUROD2, NFE2L1, NUPR1, ONECUT2, OVOL2, PATZ1, PIR, POLR3E, PRRX2, RELB, RUNX3, SIRT1, SMAD5, SNAPC5, SOX17, STRN3, SUPT6H, TBX1, TBX15, TCEA1, TCF12, TFCP2L1, TOX2, TP63, VAV1, XBP1, ZFP36L2, ZFX, ZNF143, ZNF397, ZSCAN16, ZSCAN31, NEO1, EEF1E1, EIF2S2, EIF3C/EIF3CL, EIF4EBP1, EIF1*
transport	*VPS37A, ITSN2, SVOPL, RIMS4, CACNA2D2, CACNG6, CLIC6, JPH3, KCNG1, KCNK1, KCNK5, PKD2, TRPC6, ABCB6, AP2B1, AP3S1, AP3S2, AP4S1, ATP6V0D2, CCT6B, COX18, ETFA, GOSR1, PITPNC1, SCFD1, SLC27A2, STARD3, TAP1, TIMM9, AMBP, LAMB3, SLC39A8, TC2N, FAM63B, SLC22A15, ABCC2, ABCC8, ABCG1, ATP10B, CDH23, EXOC6, MAL2, PDPN, SLC12A7, SLC22A16, SLC22A4, SLC22A5, SLC24A3, SLC30A1, SLC38A1, SLC38A2, SLC3A2, SLC47A1, SLC7A11, SLC7A5, SLCO4A1, STEAP1, STEAP2*
cell growth & proliferation	*EMILIN2, MYO5A, MYO5B, GMFB, AGT, AREG/AREGB, BMP6, BMP7, BTC, EREG, FGF18, FGF2, GDF15, IGF2, INHBB, MDK, NOV, NRG2, NRTN, NUDT6, TYMP, NGRN, PRX, GAS2L3, GAS5, CKAP4, KRT10, KRT19, MAP6, MID1IP1, MTSS1, PDLIM3, PDLIM5, RAB11FIP2, RAB11FIP4, FBLN1, FBLN2, FBN1, LAMA1, LAMA3, CABLES1, EMP1, FNDC1, FMNL1, TPD52L1, ECM2, FRAS1, LTBP1, NEBL*
cell cycle	*CCNB2, G0S2, CCNA1, CCPG1, TP53I13*

**Figure 5:**
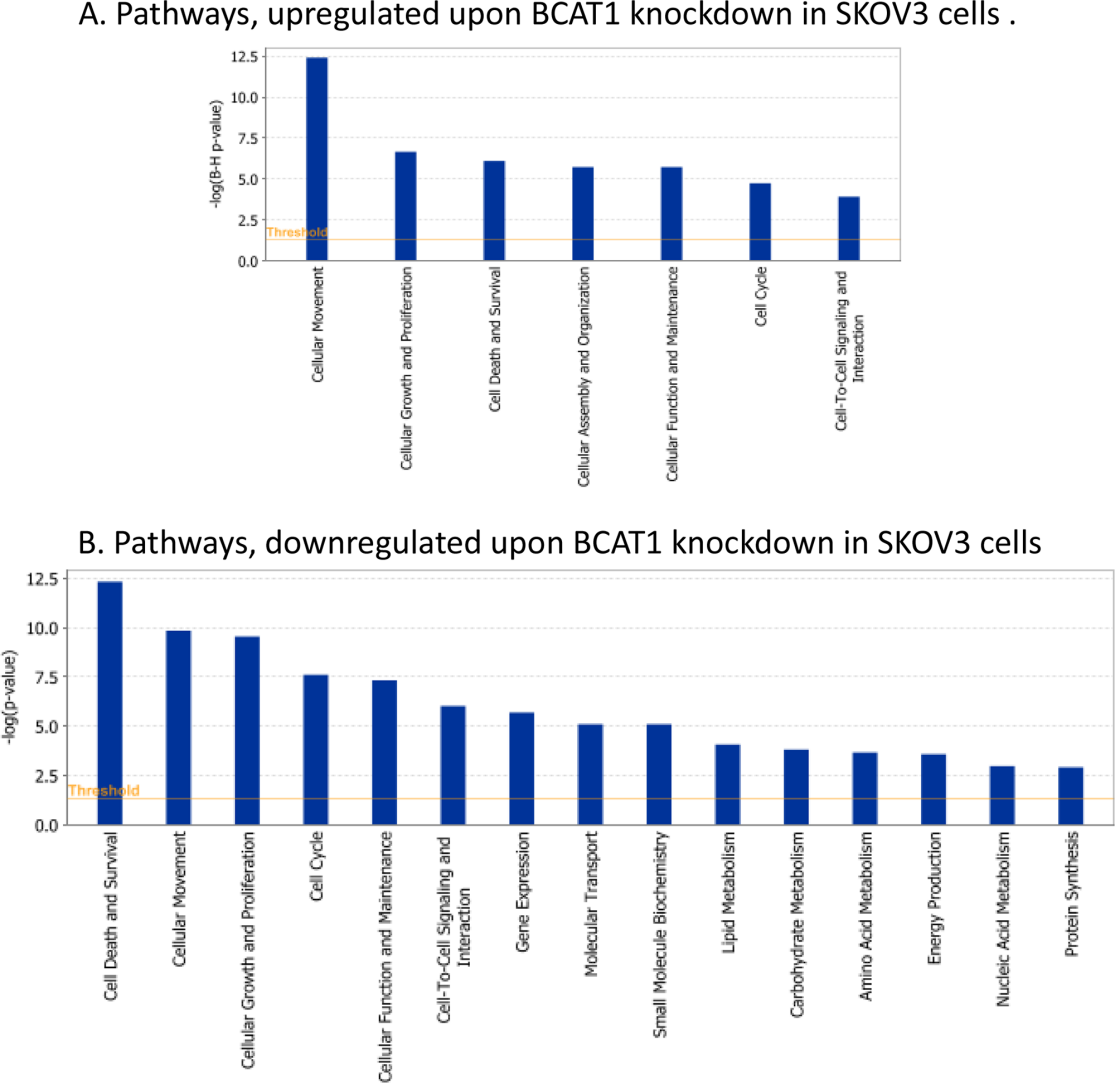
Functional analysis for a dataset of differentially expressed genes (≥ 2-fold) following BCAT1 suppression in SKOV3 cells **A.** Functional analysis of upregulated genes; **B.** Functional analysis of downregulated genes. Top functions that meet a *p*-value cutoff of 0.05 are displayed.

**Figure 6:**
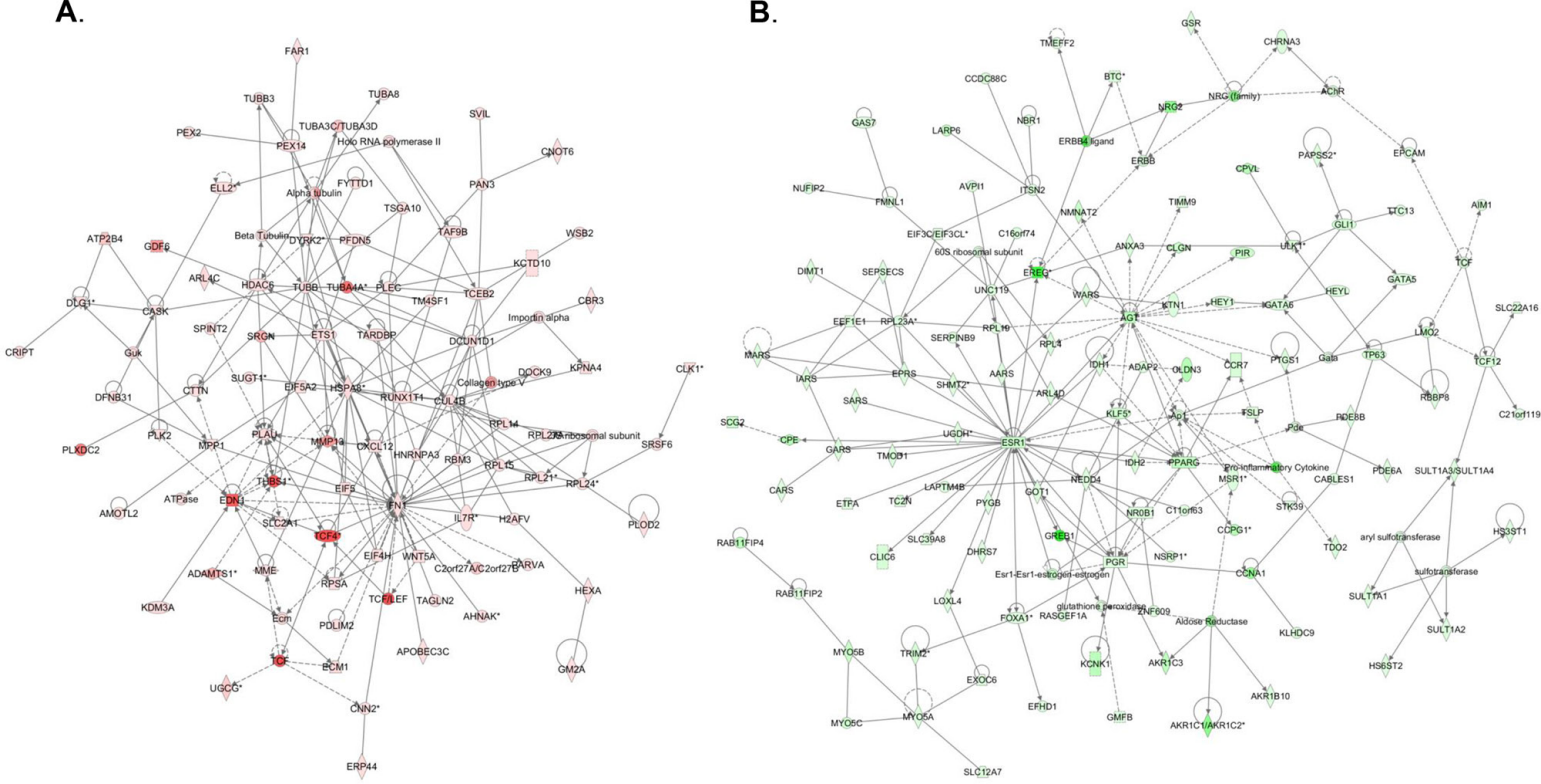
Network analysis of dynamic gene expression in SKOV3 cells based on the 2-fold common gene expression list obtained following *BCAT1* knockdown **A.** Upregulated networks; **B.** Downregulated networks. The four top-scoring networks (upregulated genes) and the five top-scoring networks (downregulated genes) were merged and are displayed graphically as nodes (genes/gene products) and edges (the biological relationships between the nodes). Intensity of the node color indicates the degree of up- (red) or downregulation (green). Nodes are displayed using various shapes that represent the functional class of the gene product (square, cytokine, vertical oval, transmembrane receptor, rectangle, nuclear receptor, diamond, enzyme, rhomboid, transporter, hexagon, translation factor, horizontal oval, transcription factor, circle, other). Edges are displayed with various labels that describe the nature of relationship between the nodes: __ binding only, → acts on. The length of an edge reflects the evidence supporting that node-to-node relationship, in that edges supported by article from literature are shorter. Dotted edges represent indirect interaction.

Pathway and network analyses, generated through the use of the IPA software confirmed the major functionally related gene groups, found to be differentially expressed in the *BCAT1* knockdown clones sh-B1 and sh-B2. As seen from Figure 5, pathways linked to cell death and survival, cellular movement, cell cycle regulation, cellular growth and proliferation, cellular function and maintenance and cell-to-cell signaling and interaction were both induced and suppressed. Notably, pathways implicated in gene expression, molecular transport, as well as carbohydrate, lipid, amino acid and nucleic acid metabolism, energy production and protein biosynthesis, were exclusively suppressed upon *BCAT1* depletion (Figure 5B). Common networks obtained upon merging the top-scoring networks recognized some important gene nodes and genes that are specifically up- or downregulated upon *BCAT1* suppression in SKOV3 cells (Figure 6). Thus, most of gene nodes that were induced upon *BCAT1* knockdown are presented in Figure 6A; they are predominantly implicated in transcription regulation (*EIF5A2, EIF5, RPSA, ETS1, HDAC6, RUNX1T1, TAF9B, TARDBP, TCEB2, TCF4*), metabolism (*CUL4B, HSPA8, PLAU, RBM3, MME, MMP13, ADAMTS1, EIF5A2, PFDN5*), cell growth and maintenance (*TUBB, MPP1, THBS1, FN1, Ecm, PLEC*), signal transduction (*EDN1, WNT5A, CXCL12*), immune response (*SRGN, IL7R*) and transport (importin alpha). The majority of gene nodes that were suppressed upon *BCAT1* knockdown in SKOV3 cells are presented in Figure 6B; these genes were mostly involved in metabolism (*PLC, IDH1, IDH2, AKR1B10, GOT1, ESR1, PTGS1, PTGS2, AKR1C3, TGM2, CPE, SULT1A3/A4*, sulfotransferase, *CDH1, RPL4, RPL19, RPL23A, NEDD4, TP63, EPRS, WARS, MARS, GARS, IARS, SARS*), signal transduction (*ANXA1, IGF2, AGT, ULK1, UNC119, NROB1, FGF2, PIK3R1, CLDN3, MSR1, Pde, PPARG, NR0B1, PGR, CCNA1, GREB1*), transcription regulation (*LMO2, GLI1, GATA6, TCF12, KLF5, FOXA1*) and cell growth and proliferation (*NRG, EREG, MYO5A, MYO5B, MYO5C*).

**Figure 7:**
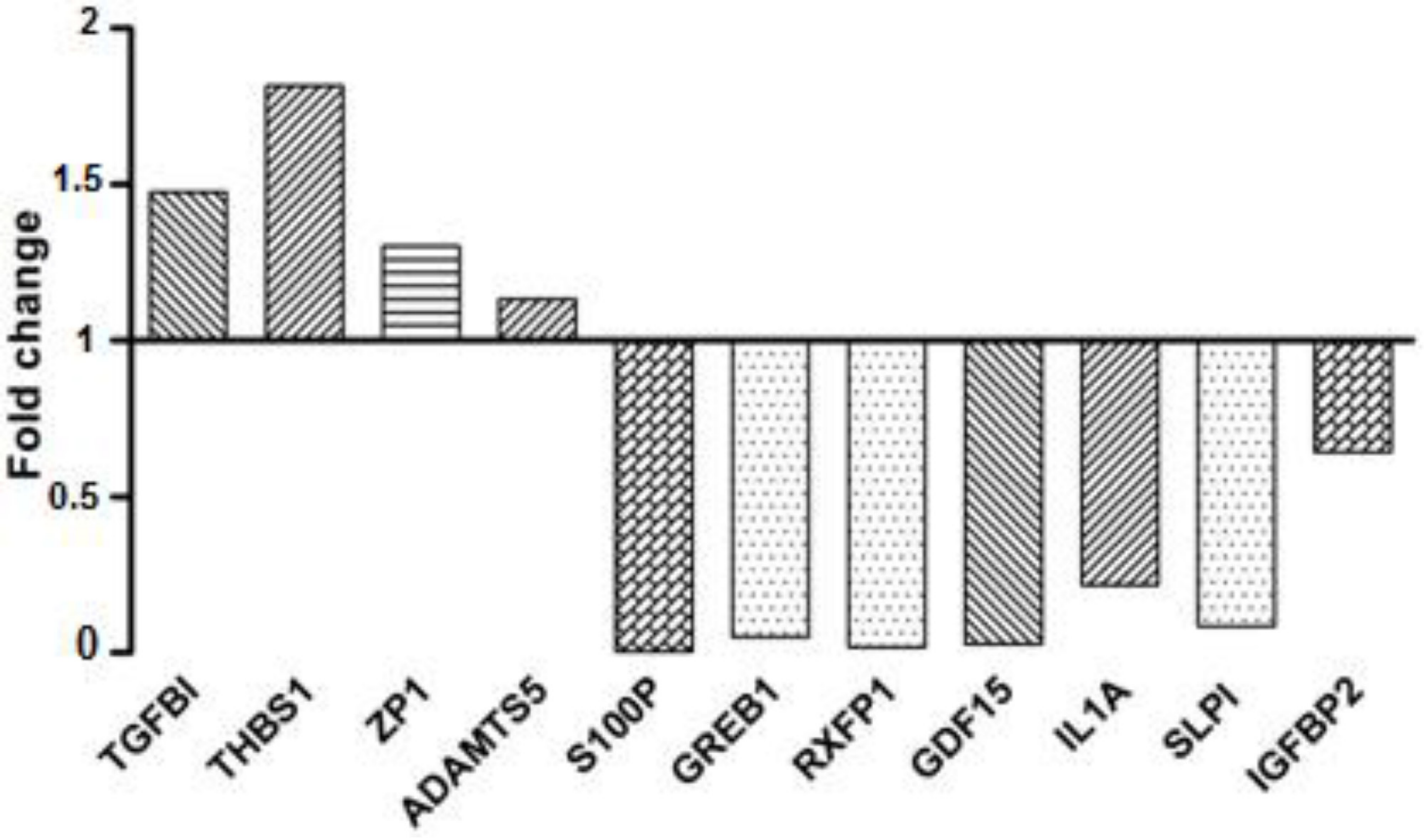
Quantitative PCR validation of microarray results The figure shows bar graphs presentation of the differential expression of the selected genes in SKOV3 cells following *BCAT1* knockdown compared to control (mock-transfected) SKOV3 cells. The relative copy number was calculated based on the target gene/18S ribosomal RNA ratio. Values more than or equal to 1 represent gene upregulation and less than 1 display gene downregulation. The analysis confirmed higher levels of expression for *TGFBI, THBS1, ZP1* and *ADAMTS5*, and lower levels of expression for *S100P, GREB1, RXFP1, GDF15, IL1A, SLP1* and *IGFBP2* upon *BCAT1* knockdown.

**Figure 8:**
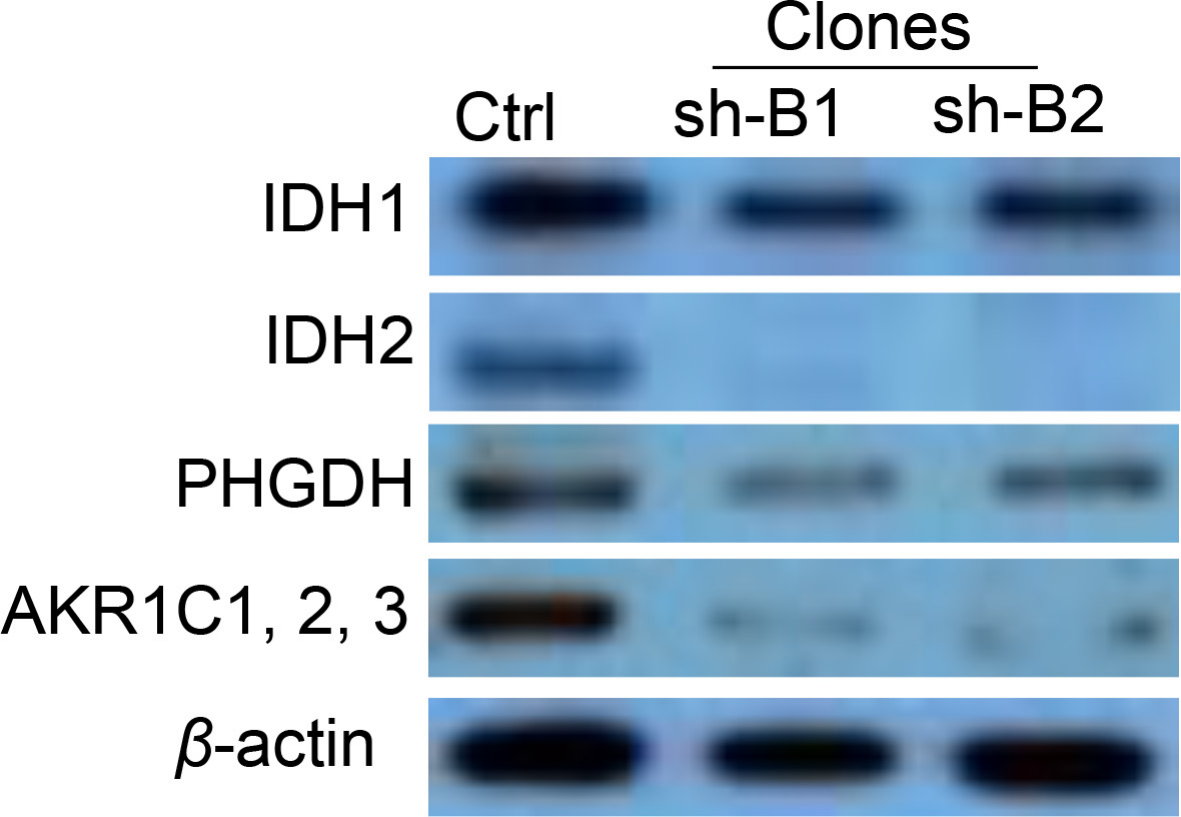
Western blot analysis of *IDH1*, *IDH2*, *PHGDH* and *AKR1C1/2/3* protein expression in *BCAT1* knockdown SKOV3 clones These proteins displayed lower expression in BCAT1 clones (sh-B1 and sh-B2), when compared to the control (Ctrl).

To validate microarray results, we arbitrarily selected 11 differentially expressed genes and quantified their expression by qPCR in SKOV3 cells following shRNA-*BCAT1* knockdown compared to control (vehicle transfected) SKOV3 cells. We found that both methods (microarray analysis and qPCR) detected similar patterns for the up- and down-regulated genes selected for validation (Figure 7). Additionally, the *BCAT1* knockdown-directed suppression of some important metabolism modulators, including *IDH1, IDH2, AKR1C1* and *PHGDH* was confirmed by Western blot analysis (see Figure 8).

### Metabolomic validation of altered metabolites’ formation in EOC cells upon *BCAT1* knockdown

By using a kit-based high-throughput flow injection mass spectroscopy (MS) approach, we analyzed the mean intracellular metabolic changes in both *BCAT1* knockdown SKOV3 clones sh-B1 and sh-B2 when compared to the Ctrl SKOV3 clone. *BCAT1* suppression resulted in lowering the concentrations of the major metabolite groups, as mean values for many amino acids, glycerophospholipids and sphingolipids were reduced to 69%, 72% and 80% respectively, when compared to the Ctrl clone (Table 3). *BCAT1* inhibition was also associated with lower concentrations of two BCAAs – Leu (69.43%) and Ile (74.31%). A significant depletion of numerous glycerophospholipids and several sphingolipids with both normal (SM) and hydroxylated SM(OH) fatty acid chains was also observed, as well as variations in some polyanines (Table 3).

**Table 3:** Mean metabolites’ values in *BCAT1* knockdown SKOV3 clones sh-B1 and sh-B2, compared to the Ctrl SKOV3 clone

Metabolites	Mean % (± SD) in *BCAT1*- KD clones sh-B1 and sh-B2 vs. the control clone^a^
***Amino acids***^1^	
Ala	72.01 ± 0.64^b^
Arg	50.10 ± 0.64
Asn	70.20 ± 1.87
Asp	78.70 ± 1.27
Glu	44.01 ± 14.78
Gly	50.00 ± 0.34
Ile	74.31 ± 0.91
Leu	69.43 ± 1.34
Lys	65.74 ± 2.39
Phe	66.36 ± 0.68
Pro	39.51 ± 2.33
Tyr	70.20 ± 0.79
**Amino acids pool**	**69.09%**
***Glycerophospholipids***^2^	
PC aa C30:0	47.62 ± 0.73
PC aa C32:1	54.05 ± 0.22
PC aa C32:2	42.41 ± 0.35
PC aa C32:3	32.83 ± 0.03
PC aa C34:1	54.13 ± 0.31
PC aa C34:2	48.13 ± 7.88
PC aa C34:3	39.05 ± 0.36
PC aa C34:4	33.62 ± 0.05
PC aa C36:2	69.68 ± 4.32
PC aa C36:3	68.89 ± 1.20
PC aa C36:4	57.70 ± 1.77
PC aa C36:5	53.51 ± 0.03
PC aa C36:6	44.47 ± 0.01
PC aa C38:0	71.81 ± 0.03
PC aa C40:2	64.65 ± 0.03
PC aa C42:5	68.69 ± 0.06
PC ae C30:0	52.62 ± 0.11
PC ae C30:1	60.79 ± 0.04
PC ae C32:1	57.79 ± 0.50
PC ae C32:2	56.82 ± 0.05
PC ae C34:1	48.39 ± 0.23
PC ae C34:2	53.81 ± 0.01
PC ae C34:3	51.57 ± 0.07
PC ae C36:1	62.10 ± 0.09
PC ae C36:2	56.09 ± 0.22
PC ae C36:3	48.63 ± 0.31
PC ae C36:4	47.37 ± 0.28
PC ae C36:5	53.38 ± 0.24
PC ae C38:2	64.17 ± 0.10
PC ae C38:3	62.70 ± 0.10
PC ae C38:4	61.80 ± 0.18
PC ae C38:5	51.21 ± 0.26
PC ae C38:6	60.48 ± 0.21
PC ae C40:2	64.49 ± 0.01
PC ae C40:3	60.70 ± 0.03
PC ae C40:4	67.78 ± 0.01
PC ae C40:5	66.57 ± 0.06
PC ae C40:6	62.58 ± 0.07
PC ae C42:4	63.32 ± 0.03
PC ae C44:6	45.19 ± 0.01
**PC pool**	**72.00%**
***Sphingolipids***^3^	
SM (OH) C14:1	75.99 ± 0.08
SM (OH) C16:1	67.81 ± 0.01
SM (OH) C22:2	72.10 ± 0.09
SM C16:0	72.90 ± 1.21
SM C16:1	74.62 ± 0.59
SM C18:0	77.05 ± 0.04
SM C18:1	71.02 ± 0.23
SM C24:0	70.19 ± 0.10
SM C24:1	69.24 ± 0.41
SM C26:1	65.51 ± 0.03
**SM pool**	**79.65%**
***Acylcarnitines***^4^	
C14:1	70.02 ± 0.00
***Biogenic amines***^5^	
Ac-Orn	45.81 ± 0.01
Putrescine	527.34 ± 0.01
Sarcosine	69.77 ± 1.13

a Percentage are derived from medium values for both *BCAT1* knockdown clones (shB1 & sh-B2)

b Standard Deviation (SD) values are displayed

1 Ala: Alanine; Arg: Arginine; Asn: Asparagine; Asp: Aspartic acid; Glu:Glutamic acid; Gly :Glycine; Ile: Isoleucine; Leu: leucine; Lys: Lysine; Phe: Phenylalanine; Pro: Proline; Ser: Serine; Thr: Threonine; Tyr: Tyrosine.

2 PC aa: phosphatidylcholine diacyl; PC ae: phosphatidylcholines acyl-alkyl

3 SM (OH): Hydroxysphingomyeline; SM:sphingomyeline

4 Tetradecenoylcarnitine

5 Ac-Orn: Acetyl-ornithine

### *BCAT1* suppression inhibits tumor expansion and metastasis in nude mice

**Figure 9:**
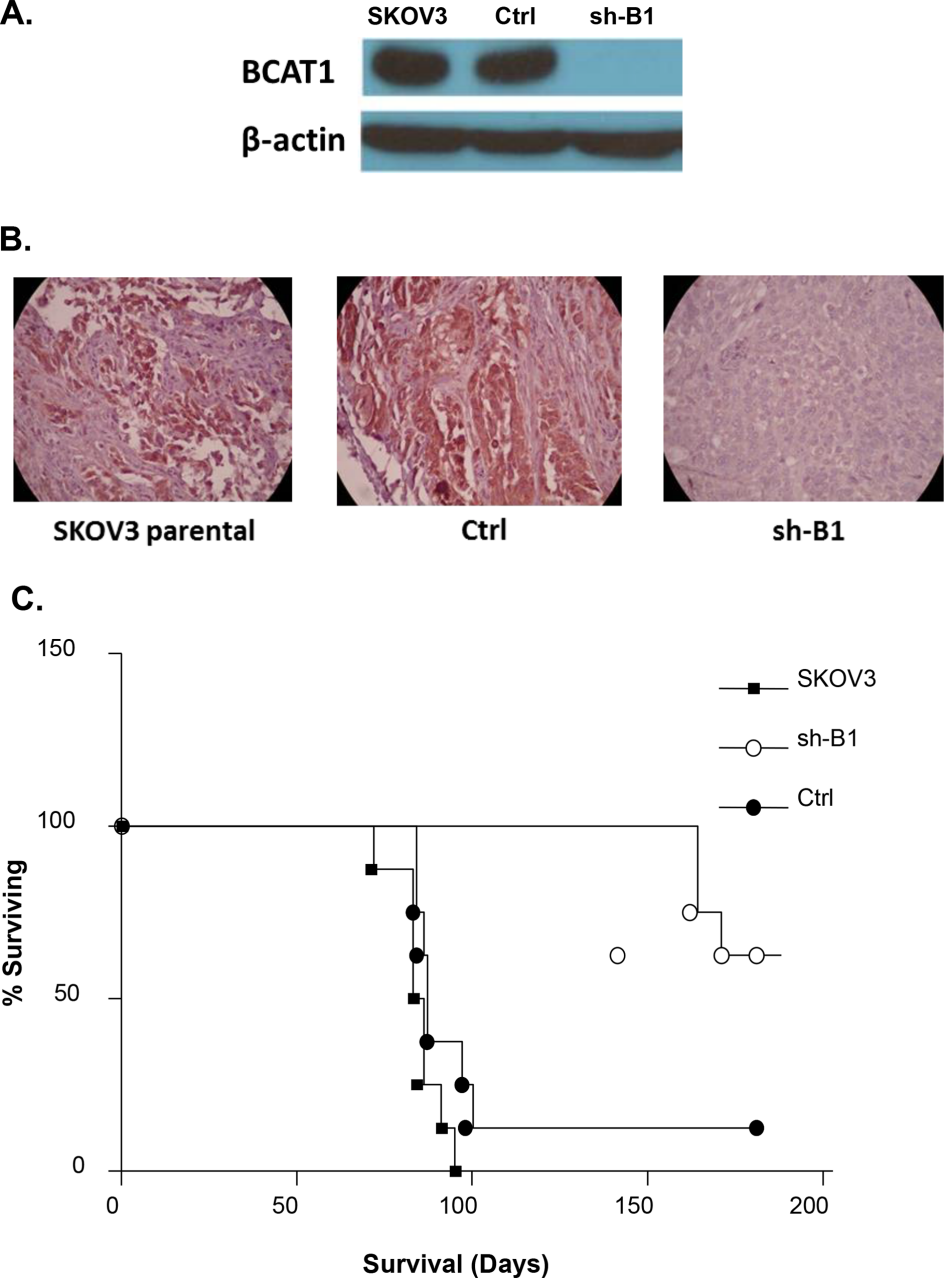
A. Western-blot analysis of *BCAT1* protein expression in tumor tissues extracted from nude mice, injected with the parental SKOV3 cells, the mock-transfected (Ctrl) cells, and the BCAT1 knockdown (sh-B1 cells **B.** Representative IHC images of *BCAT1* expression in tumor tissues extracted from nude mice, injected with the parental SKOV3 cells, Ctrl cells, and sh-B1 cells. **C.** Survival curves for mice injected with parental, Ctrl, and sh-B1 SKOV3 cells. The median survival of mice injected with the Ctrl cells (87 days, *n* = 6) is similar to the parental SKOV3 cells (85 days, *n* = 7). Survival of mice injected with the *BCAT1* knockdown cells is significantly longer than the vector control (165 days; *P* = 0.0031, Log rank test), with 5 mice still alive more than 200 days after injection with the *BCAT1* knockdown cells.

In order to investigate the effect of *BCAT1* knockdown on EOC dissemination *in vivo*, intact SKOV3 cells, as well as mock (Ctrl) and shRNA-*BCAT1* (clone sh-B1) transduced SKOV3 cells were injected intraperitoneally (IP) in nude mice (*n* = 6–7 per experimental group). The *BCAT1* suppression in tumors of mice injected with the *BCAT1*-knockdown cells was confirmed by Western blot and IHC staining (Figures 9A and 9B). Moreover, global gene expression comparison in tumor tissues extracted from two nude mice i.p. injected with sh-B1 cells, versus tumors from two mice injected with Ctrl cells, displayed evident similarity between the major functionally related gene groups that were differentially expressed upon *BCAT1* silencing both *in vitro* and *in vivo* (see Figure 5 and Supplemental Figure 5).

The total tumor burden and the ascites volume were not different among the three experimental groups (data not shown). However, *BCAT1* knockdown had a major impact on survival. While the median survival of mice injected with the vector-control cells (87 days, *n* = 6) was similar to the parental SKOV3 cells (85 days, *n* = 7), the survival of mice injected with *BCAT1* knockdown cells was significantly longer than the vector-control (165 days; *p* = 0.0031, Logrank test), with 5 mice still alive more than 200 days after injection with the *BCAT1* knockdown cells (Figure 9C).

### The transcription factor *c-Myc* regulates *BCAT1* expression in EOC cells

**Figure 10:**
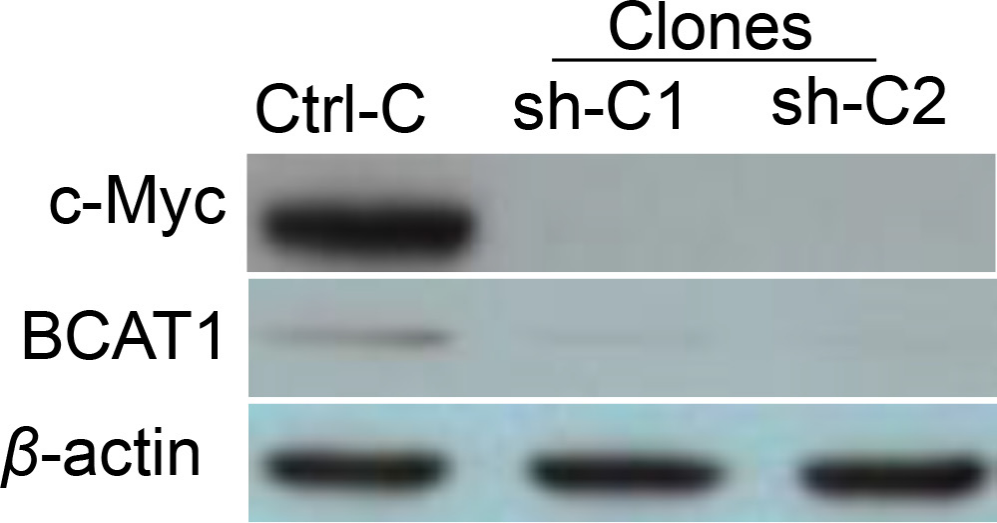
Western blot analysis of *BCAT1* protein expression in shRNA-mediated c-*Myc* knockdown clones sh-C1 and sh-C2, compared to the control (Ctrl-C) clone

*c-Myc* represents a major oncogene, frequently overexpressed in EOC [18]. *BCAT1* has been previously identified as one of the *c-Myc* target genes in different cancers [15, 19, 20]; however, the *c-Myc-BCAT1* link has been not studied in EOC. To investigate the regulation of *BCAT1* by *c-Myc* in EOC cells, shRNA-related *c-Myc* knockdown was performed in SKOV3 cells. Clone selection for further analyses was based on Western blot validation of the *c-Myc* protein expression in selected clones, compared to empty vector-transfected clones. Among the analyzed clones, the shRNA-*c-Myc* knockdown clones sh-C1 and sh-C2 displayed a significant decrease of *c-Myc* protein expression levels compared to the mock-transfected control (Ctrl) (Figure 10); as expected, the c-Myc knockdown resulted in evident suppression of the *BCAT1* protein expression (Figure 10).

## DISCUSSION

We have recently identified a 425 nt CpG-rich region of the *BCAT1* gene that is significantly hypomethylated in HG serous EOC tumors, compared to normal tissue [8]. This region is positioned within the first intron of *BCAT1* gene isoforms I, II and III; however, it covers a potential control (promoter 2) region for *BCAT1* isoforms IV and V. Indeed, the 425 nt hypomethylated region spans the transcription initiation site (−105 nt to +320 nt) of *BCAT1* gene isoform IV, and is located upstream of the transcription initiation site (−370 nt to −795 nt) of *BCAT1* gene isoform V (comprising CpG island 70 - see Supplemental Figure 6). Since all five *BCAT1* isoforms share highly homologous amino acids sequences (ranging from 89% to 99%), our findings confirm the previously proposed role of DNA methylation in controlling alternative promoter usage, both during normal cell differentiation and neoplastic transformation [16, 21]. The implication of aberrant DNA methylation in the control of *BCAT1* expression in cancer has been also demonstrated in gliomas and colorectal cancer [16, 21]. These findings suggest that epigenetic mechanisms could account for altered *BCAT1* expression in different cancer types, including EOC.

Moreover, our functional analyses are strongly indicative for evident oncogenic capacity of *BCAT1* in EOC, including its potential implication in EOC cell proliferation, cell cycle control and cell migration/invasion (see Figures 2, 3, 4), in concordance with similar findings about the role of *BCAT1* in other cancers [12–16]. The consecutive gene expression experiments and related network and pathway analyses were quite confirmatory of the data obtained by the *BCAT1* functional assays (see Table 2 and Figure 5).

IPA network analysis was indicative for some important gene nodes linked to *BCAT1* suppression in EOC cells, as most of these substantiate and/or complement the functional data obtained. As shown on Figure 6A, *BCAT1* knockdown resulted in upregulation of a number of gene nodes with previously suggested TSG function in EOC, including *RBM3, THBS1, RUNX1T1* and *WNT5A* [22–25], as well as some TSGs, characterized in other cancer types (*ADAMTS1, PFDN5/MM-1, MME, MPP1, TCEB2*) [26–30]. Other induced gene nodes comprised genes, known in promoting cellular death/apoptosis, such as *ETS1, SRGN, TCF4* and *CUL4B* [31–34].

*BCAT1* suppression in SKOV3 cells led to the induction of several gene nodes (*EIF5A2, EIF5* and *EIF4H*), as part of the eukaryotic initiation factors (eIFs) network, shown to display all elements of a complete oncogenic, as well as tumor suppressive cascade [35]. Similarly, some of the induced genes nodes upon *BCAT1* knockdown (*PLEC, HDAC6, CXCL12, DCUN1D1/SCCRO*) have exhibited dual functions in tumorigenesis [36–43]. Some known oncogenes (*MMP13, FN, EDN1* and *PLAU*) [44–47] also displayed increased expression upon *BCAT1* knockdown, which could be indicative for some compensatory carcinogenic mechanisms in SKOV3 cells following *BCAT1* suppression.

In parallel, upon *BCAT1* knockdown we have observed substantial downregulation of major gene nodes known to be implicated in EOC tumorigenesis (*PPARG, ANXA3, TGM2, Ap1/c-jun, NRG & NRG2, TP63, PGR, MSR1, PIK3R1, KLF5, TCF*) [48–58], including EOC dissemination/metastasis (*CLDN3, EPCAM, PTGS1/COX1 & PTGS2/COX2, ESR1, GLI1, GREB1, ERBB, IGF2, FGF2, CDH1*) [59–69] (Figure 6B). The *BCAT1* silencing in SKOV3 cells also resulted in suppression of numerous gene nodes with proven oncogenic potential in different cancers (including *EREG, RPL23A, LMO2, TCF12, FMNL1, MYO5A, CCR7, NEDD4, ANXA1, TSLP, RPL4, AGT, FOXA1, CPE, RPL19*) [70–84] (Figure 6B). Thus, our findings are strongly indicative for the oncogenic functionality of *BCAT1* in EOC etiology.

Moreover, *BCAT1* suppression was notably associated with downregulation of numerous metabolism-related gene nodes, predominantly implicated in regulation of lipid metabolism (*PLC, NROB1, AKR1B10, SULT1A3/A4, GOT1, ESR1, PGR, MSR1, ANXA1, PTGS1, PTGS2, AGT, KLF5, PIK3R1, MYO5A, AKR1C3, FOXA1, PPARG*) (Figure 6B). Among these, sulfotransferases (*SULT1A1, SULT1A2, SULT1A3*) and aldo-keto reductases (*AKR1C1, AKR1C2, AKR1C3*) were shown to contribute to disease progression and/or represent therapeutic targets in hormone-dependent forms of cancer [85, 86], including EOC [87].

A number of aminoacyl-tRNA synthetases (ARSs), including *EPRS, MARS, SARS, WARS, IARS, GARS, AARS*, were suppressed upon *BCAT1* silencing in EOC cells. These enzymes play a critical role in protein biosynthesis by charging tRNAs with their cognate amino acids; moreover, recent findings are indicative the potential pathophysiological implications of ARSs in tumorigenesis [88].

The two isoforms of the isocitrate dehydrogenase enzyme (*IDH1* and *IDH2*) were also among the major gene nodes that were suppressed upon *BCAT1* silencing (see also Figure 6B). The *IDH1* and *IDH2* enzymes catalyze the oxidative decarboxylation of isocitrate to α-ketoglutarate, generating NADPH from NADP+. *IDH* enzymes are associated with the tricarboxylic acid (TCA) cycle for energy production and are thereby involved in multiple metabolic processes [89]. Mutations of the *IDH1/2* genes represent significant driver mutations in gliomas and acute myeloid leukemia development, but are quite rare/almost undetectable in solid tumors [90]. Recent study has demonstrated a link between *IDH1* function and *BCAT1* expression in gliomas, as *BCAT1* was shown to be involved in tumor growth and disease progression only in gliomas with wild-type (non-mutated) *IDH1* [16]. Moreover, shRNA-directed knockdown of *IDH1* led to strong downregulation of *BCAT1* expression in glioblastoma cell lines [16]. Our data are indicative for the inverse relationship regarding the impact of the *BCAT1* expression on the expression of the *IDH* genes. Additionally, the aldo-keto reductase A*KR1C1* and the phosphoglycerate dehydrogenase (*PHGDH*) were among the most strongly suppressed genes (−15.20 and −14.58, respectively) in both (sh-B1 and sh-B2) *BCAT1* knockdown clones (see Suppl. Table 1B). Both these genes are actively implicated in cellular metabolism and have been previously characterized as therapeutic cancer targets. Indeed, *AKR1C1* was shown to induce resistance to cisplatin in human EOC cells [91] and was thus recognized as potential EOC therapeutic target [87]. *PHGDH* catalyzes the first step in the serine biosynthesis pathway by diverting glucose-derived carbon into serine and glycine metabolism. Recently, the essential role of the serine biosynthesis pathway in metabolic reprogramming in cancer has been recognized, and *PHGDH* was found to be overexpressed and to contribute to tumor cell growth in a significant subset of human cancers [92, 93]. The *IDH1, IDH2, ACR1C1* and *PHGDH* downregulation following *BCAT1* knockdown was also confirmed on the protein level (see Figure 8).

Therefore, *BCAT1* silencing could exert multiple inhibitory effects on the TCA cycle-associated energy production, and could impact EOC metabolism by suppressing lipid production and protein synthesis, including the serine biosynthesis pathway. This was further supported by our initial and rather exploratory metabolomic investigation. Indeed, the *BCAT1* knockdown phenotype was associated with significant depletion of most amino acids and numerous phospho- and sphingolipids, as well as with perturbations in metabolism of some polyamines.

EOC spreads by intraperitoneal (IP) sloughing, lymphatic invasion, and hematogenous dissemination [94]. IP dissemination is the most common; after malignant cells have evaded from the ovarian capsule, they are shed from the tumor surface into the peritoneal cavity where they follow normal routes of peritoneal fluid [95]. Hence, IP injection of cancer cells in animal models can accurately model advanced disease, as EOC metastases frequently appear disseminated throughout the peritoneum [96]. We used a similar *in vivo* approach to investigate the role of *BCAT1* in EOC progression. We found that SKOV3 cells dissemination was strongly abrogated following *BCAT1* knockdown, which led to significant increase of survival rate in animals treated with *BCAT1* knockdown cells.

In conclusion, we have shown that *BCAT1* is significantly overexpressed in LMP tumors and HG serous EOC tumors compared to normal ovarian tissues, as epigenetic mechanisms (DNA hypomethylation) could be involved in *BCAT1* overexpression in this tumor type. We also confirmed that *c-Myc* regulates *BCAT1* expression in EOC cells. Functional analyses pointed towards *BCAT1* implication in the control of EOC cell proliferation (including cell cycle control), migration and invasion. Gene expression profiling and associated network and pathway analyses confirmed these findings, as numerous genes and pathways known previously to be implicated in ovarian tumorigenesis, including EOC tumor invasion and metastasis, were found to be suppressed upon *BCAT1* knockdown, while some TSGs were induced. Importantly, our microarray data and consecutive metabolomics analyses are indicative for a major *BCAT1* implication in controlling EOC metabolism. Finally, *BCAT1* knockdown led to reduced dissemination and prolonged survival time in xenograft models of advanced peritoneal EOC. Our data provide both *in vitro* and *in vivo* proof-of-concept that *BCAT1* represents a major EOC oncogene and suggest that *BCAT1* should be further explored as a potential EOC therapeutic target.

## MATERIALS AND METHODS

### Patients and tissue specimens

Snap frozen and formalin-fixed paraffin-embedded (FFPE) tissues of 117 EOC tumors were obtained at the Hotel-Dieu de Quebec Hospital, Quebec, Canada. These included 13 borderline, or LMP tumors and 104 HG adenocarcinomas. None of the patients received CT before surgery (see Table 1 for detailed clinicopathological characteristics). All tumors were histologically classified according to the criteria defined by the World Health Organization [97]. The CT treatment was completed for all patients and the response to treatment was known. Disease progression was evaluated following the guidelines of the Gynecology Cancer Intergroup [97]. PFS was defined as the time from surgery to the first observation of disease progression, recurrence or death. Thirteen normal ovarian samples were derived from women subjected to hysterectomy with oophorectomy due to non-ovarian pathologies. The study was approved by the Clinical Research Ethics Committee of the Hotel-Dieu de Quebec Hospital and all patients signed an informed consent for voluntary participation.

### Cell cultures

The EOC cell lines OVCAR3 and SKOV3 were purchased from American Tissue Type Collection (Manassas, VA); OV-90, OV2008, TOV-112 and TOV-21 cell lines were a kind gift from Dr. Anne-Marie Mes-Masson (Université de Montréal), while A2780s and A2780cp cell lines were a kind gift from Dr. Benjamin Tsang (University of Ottawa). The cell lines were passed in different culture media supplemented with 10% fetal bovine serum, as previously described [98].

### Tissue microarrays (TMAs) construction and immunohistochemistry (IHC)

TMAs construction and IHC analyses were performed as previously described [98–100]. Briefly, one representative 4 μm tissue section was cut from the tissue array blocks. Sections were deparaffinized and rehydrated in graded alcohols, then incubated with blocking serum for 20 min. Sections were incubated overnight at room temperature with the *BCAT1* rabbit polyclonal antibody (Proteintech) at dilution 1:50. Sections were then incubated with a biotinylated secondary antibody (Dako, Carpinteria, CA) and then exposed to a streptavidin complex (Dako, Carpinteria, CA). Complete reaction was revealed by 3–3 diaminobenzidine and slides were counterstained with hematoxylin.

*BCAT1* protein expression was assessed by semi-quantitative scoring of the intensity of staining and recorded as absent (0), weak (1+), moderate (2+) or strong (3+). The IHC staining was analyzed independently by 2 pathologists blinded to clinical data and progression. The relationship between *BCAT1* expression in serous ovarian carcinomas and normal ovarian tissues was evaluated by the Wilcoxon two-sample test. A significant association was considered when *p*-value was below 0.05. A Kaplan-Meier curve and the log-rank test were performed based on PFS values to test the effect of the intensity of *BCAT1* (3, 2 versus 0, 1) on disease progression.

### Short Hairpin RNA (shRNA) – mediated *BCAT1* and *c-Myc* knockdowns in SKOV3 and A2780s cells

A shRNA, targeting the *BCAT1* sequence 5′-GAAGAACTGGCAACTCCTC-3′, was designed using the siRNA Ambion Target Finder software (http://www.ambion.com/techlib/misc/siRNA_finder.html), and subcloned into the pSilencer 4.1 puro vector (Ambion). SKOV3 cells were stably transfected with the shRNA-*BCAT1* plasmid using the ExGen 500 transfection reagent (Fermentas Canada Inc., Burlington ON), according to the manufacturer's instructions. Cells were consecutively grown for 2 weeks in selection medium containing 0.5 μg/ml puromycin (Wisent, Canada) to isolate stable clones. Cells were also mock-transfected with the pSilencer 4.1 puro vector, and the stably-transfected clones were isolated as controls. Stable clones with inhibited *BCAT1* expression were evaluated and validated by semi-quantitative RT-PCR and Western blot, as previously described [101].

A *c-Myc* shRNA cloned into the pLKO.1-puro vector was retrieved from the Sigma Mission TRC human 1.5 shRNA library (clone number TRCN0000039642). Viral supernatants were generated by transfecting 293T cells with the shRNA construct and the packaging vectors psPAX2 and pMD2.G (Addgene, Cambridge, MA). The high-titer lentiviral supernatants in the presence of 5 μg/ml polybrene were used to infect SKOV3 cells. Two days later, infected cells were treated with puromycin (0,5 μg/ml) for the selection of stably-transduced clones. The pLKO.1-puro vector encoding a scramble sequence not matching any mammalian sequence was used for the generation of mock-transduced (Ctrl-C) clones. Stable clones with inhibited *c-Myc* expression were evaluated and validated by Western blot.

### Functional assays

Different functional assays, including real time cell proliferation monitoring (using the xCELLigence Real-Time Cell Analyzer), colony formation, cell cycle analysis, cell migration and invasion assays and cell cytotoxity (MTT) assays were performed as previously shown [98, 101, 102].

### Gene expression profiling and data analysis

Gene expression profiling and microarray analysis was carried out using the Agilent Whole Human Genome microarrays, containing 44,000 genes, as described before [98, 100, 102]. Network analysis of the microarray data was completed using the Ingenuity Pathway Analysis (IPA) software (see http://www.Ingenuity.com). The microarray data have been deposited to the GEO database (http://www.ncbi.nlm.nih.gov/geo/) with accession number GSE64424. Selected microarray data were confirmed by quantitative PCR (qPCR) as described previously [102]. The primers used for sqRT-PCR and qPCR validation are listed in Supplemental Table 2.

### Western blotting

Western blot analysis was performed as previously described [98, 100, 102]. Antibodies used for monitoring protein expression were the anti-*BCAT1* rabbit polyclonal Ab (1:500) (Proteintech), the anti-*IDH1* goat polyclonal Ab (1:500) (Santa Cruz Biotechnology), the anti-*IDH2* mouse monoclonal Ab (1:500) (Santa Cruz Biotechnology), the anti-*PHGDH* mouse polyclonal Ab (1:500) (Santa Cruz Biotechnology), the anti-*AKR1C1, 2, 3* goat polyclonal Ab (1:500) (Santa Cruz Biotechnology), and the anti-β-actin Ab (1:5000) (Santa Cruz Biotechnology), the latter used as internal standard. The anti-*c-Myc* Ab was a gift from Dr. Gobeil's lab.

### Metabolomics analyses

5 × 10^6^ SKOV3 cells were plated in 75 cm flasks and grown for 48 h before counting and collecting the cells by trypsinization. Upon 2 × washing with cold PBS, cells were lysed by adding 10 mM phosphate buffer, pH 7.4 (15% v/v) and cold 100% EtOH (85% v/v). Lysed cell suspensions were subjected to three rounds of sonication-freeze-thaw to further ensure cell disruption and protein denaturation and centrifuged at 20000 g for 10 minutes at 4°C to remove any pellet. Supernatants were transferred into cryogenic vials, and stored at −160°C.

Metabolomics analysis was performed using a commercially available assay using the LC-MS/MS based AbsoluteIDQ p180 Kit (Biocrates Life Sciences AG, Austria), which allows the quantification of up to 188 endogenous metabolites from 5 different compound classes including acylcarnitines, amino acids, hexoses, phospho- and sphingolipids and biogenic amines [103]. MS analyses were done on a API 4000 LC/MS/MS System (AB Sciex, Concord, ON, Canada) equipped with a UFLC Prominence (Shimadzu Scientific Instrument Inc., Columbia, MD, USA) and controlled by the software Analyst version 1.6.2. Isotope-labeled internal standards and other internal standards are integrated into a kit plate filter for metabolite quantification. The Biocrates MetIDQ software was used to control the assay workflow, from sample registration to automated calculation of metabolite concentrations and the export of data into other data analysis programs. The metabolomics analyses were performed at the Pharmacogenomics laboratory, CHU Research Center, Québec, Canada (http://www.pharmacogenomics.pha.ulaval.ca/).

### Peritoneal tumor formation in mice

Intact SKOV3 cells, as well as the SKOV3 sh-B1 clone and the SKOV3 Ctrl clone (1 × 10^7^ cells in 500 μL of PBS), were IP injected into 8 × 8 week old CB17 SCID female mice (CB17/Icr-Prkdcscid/IcrIcoCrl strain code 236, Charles River) using a 25G5/8 needle. Mice were monitored daily by staff blinded to the cell type injected and euthanized when they reached a loss of wellness endpoint that was most often respiratory distress associated with ascites accumulation. The animals had free access to food and water and experiments were done in accordance with the Canadian Council on Animal Care's Guidelines for the Care and Use of Animals. Protocols were approved by the University of Ottawa Animal Care Committee.

## SUPPLEMENTARY FIGURES AND TABLES





## References

[1] Jemal A, Siegel R, Xu J, Ward E (2010). Cancer statistics, 2010. CA: a cancer journal for clinicians.

[2] Marchetti C, Pisano C, Facchini G, Bruni GS, Magazzino FP, Losito S, Pignata S (2010). First-line treatment of advanced ovarian cancer: current research and perspectives. Expert review of anticancer therapy.

[3] Jones PA, Baylin SB (2007). The epigenomics of cancer. Cell.

[4] Balch C, Fang F, Matei DE, Huang TH, Nephew KP (2009). Minireview: epigenetic changes in ovarian cancer. Endocrinology.

[5] Bauerschlag DO, Ammerpohl O, Brautigam K, Schem C, Lin Q, Weigel MT, Hilpert F, Arnold N, Maass N, Meinhold-Heerlein I, Wagner W (2011). Progression-free survival in ovarian cancer is reflected in epigenetic DNA methylation profiles. Oncology.

[6] Watts GS, Futscher BW, Holtan N, Degeest K, Domann FE, Rose SL (2008). DNA methylation changes in ovarian cancer are cumulative with disease progression and identify tumor stage. BMC medical genomics.

[7] Li M, Balch C, Montgomery JS, Jeong M, Chung JH, Yan P, Huang TH, Kim S, Nephew KP (2009). Integrated analysis of DNA methylation and gene expression reveals specific signaling pathways associated with platinum resistance in ovarian cancer. BMC medical genomics.

[8] Keita M, Wang ZQ, Pelletier JF, Bachvarova M, Plante M, Gregoire J, Renaud MC, Mes-Masson AM, Paquet ER, Bachvarov D (2013). Global methylation profiling in serous ovarian cancer is indicative for distinct aberrant DNA methylation signatures associated with tumor aggressiveness and disease progression. Gynecologic oncology.

[9] Hall TR, Wallin R, Reinhart GD, Hutson SM (1993). Branched chain aminotransferase isoenzymes. Purification and characterization of the rat brain isoenzyme. The Journal of biological chemistry.

[10] Eden A, Simchen G, Benvenisty N (1996). Two yeast homologs of ECA39, a target for c-Myc regulation, code for cytosolic and mitochondrial branched-chain amino acid aminotransferases. The Journal of biological chemistry.

[11] Schuldiner O, Eden A, Ben-Yosef T, Yanuka O, Simchen G, Benvenisty N (1996). ECA39, a conserved gene regulated by c-Myc in mice, is involved in G1/S cell cycle regulation in yeast. Proceedings of the National Academy of Sciences of the United States of America.

[12] Weggen S, Preuss U, Pietsch T, Hilger N, Klawitz I, Scheidtmann KH, Wiestler OD, Bayer TA (2001). Identification of amplified genes from SV40 large T antigen-induced rat PNET cell lines by subtractive cDNA analysis and radiation hybrid mapping. Oncogene.

[13] Rodriguez S, Jafer O, Goker H, Summersgill BM, Zafarana G, Gillis AJ, van Gurp RJ, Oosterhuis JW, Lu YJ, Huddart R, Cooper CS, Clark J, Looijenga LH, Shipley JM (2003). Expression profile of genes from 12p in testicular germ cell tumors of adolescents and adults associated with i(12p) and amplification at 12p11.2-p12.1. Oncogene.

[14] Altmaier E, Kastenmuller G, Romisch-Margl W, Thorand B, Weinberger KM, Illig T, Adamski J, Doring A, Suhre K (2011). Questionnaire-based self-reported nutrition habits associate with serum metabolism as revealed by quantitative targeted metabolomics. European journal of epidemiology.

[15] Zhou W, Feng X, Ren C, Jiang X, Liu W, Huang W, Liu Z, Li Z, Zeng L, Wang L, Zhu B, Shi J, Liu J, Zhang C, Liu Y, Yao K (2013). Over-expression of BCAT1, a c-Myc target gene, induces cell proliferation, migration and invasion in nasopharyngeal carcinoma. Molecular cancer.

[16] Tonjes M, Barbus S, Park YJ, Wang W, Schlotter M, Lindroth AM, Pleier SV, Bai AH, Karra D, Piro RM, Felsberg J, Addington A, Lemke D, Weibrecht I, Hovestadt V, Rolli CG (2013). BCAT1 promotes cell proliferation through amino acid catabolism in gliomas carrying wild-type IDH1. Nature medicine.

[17] Baracos VE, Mackenzie ML (2006). Investigations of branched-chain amino acids and their metabolites in animal models of cancer. The Journal of nutrition.

[18] Aunoble B, Sanches R, Didier E, Bignon YJ (2000). Major oncogenes and tumor suppressor genes involved in epithelial ovarian cancer (review). International journal of oncology.

[19] Ben-Yosef T, Yanuka O, Halle D, Benvenisty N (1998). Involvement of Myc targets in c-myc and N-myc induced human tumors. Oncogene.

[20] Ben-Yosef T, Eden A, Benvenisty N (1998). Characterization of murine BCAT genes: Bcat1, a c-Myc target, and its homolog, Bcat2. Mammalian genome: official journal of the International Mammalian Genome Society.

[21] Mitchell SM, Ross JP, Drew HR, Ho T, Brown GS, Saunders NF, Duesing KR, Buckley MJ, Dunne R, Beetson I, Rand KN, McEvoy A, Thomas ML, Baker RT, Wattchow DA, Young GP (2014). A panel of genes methylated with high frequency in colorectal cancer. BMC cancer.

[22] Ehlen A, Nodin B, Rexhepaj E, Brandstedt J, Uhlen M, Alvarado-Kristensson M, Ponten F, Brennan DJ, Jirstrom K (2011). RBM3-regulated genes promote DNA integrity and affect clinical outcome in epithelial ovarian cancer. Translational oncology.

[23] Alvarez AA, Axelrod JR, Whitaker RS, Isner PD, Bentley RC, Dodge RK, Rodriguez GC (2001). Thrombospondin-1 expression in epithelial ovarian carcinoma: association with p53 status, tumor angiogenesis, and survival in platinum-treated patients. Gynecologic oncology.

[24] Yeh KT, Chen TH, Yang HW, Chou JL, Chen LY, Yeh CM, Chen YH, Lin RI, Su HY, Chen GC, Deatherage DE, Huang YW, Yan PS, Lin HJ, Nephew KP, Huang TH (2011). Aberrant TGFbeta/SMAD4 signaling contributes to epigenetic silencing of a putative tumor suppressor, RunX1T1 in ovarian cancer. Epigenetics: official journal of the DNA Methylation Society.

[25] Bitler BG, Nicodemus JP, Li H, Cai Q, Wu H, Hua X, Li T, Birrer MJ, Godwin AK, Cairns P, Zhang R (2011). Wnt5a suppresses epithelial ovarian cancer by promoting cellular senescence. Cancer research.

[26] Martino-Echarri E, Fernandez-Rodriguez R, Rodriguez-Baena FJ, Barrientos-Duran A, Torres-Collado AX, Plaza-Calonge Mdel C, Amador-Cubero S, Cortes J, Reynolds LE, Hodivala-Dilke KM, Rodriguez-Manzaneque JC (2013). Contribution of ADAMTS1 as a tumor suppressor gene in human breast carcinoma. Linking its tumor inhibitory properties to its proteolytic activity on nidogen-1 and nidogen-2. International journal of cancer Journal international du cancer.

[27] Fujioka Y, Taira T, Maeda Y, Tanaka S, Nishihara H, Iguchi-Ariga SM, Nagashima K, Ariga H (2001). MM-1, a c-Myc-binding protein, is a candidate for a tumor suppressor in leukemia/lymphoma and tongue cancer. The Journal of biological chemistry.

[28] Meng F, Demorrow S, Venter J, Frampton G, Han Y, Francis H, Standeford H, Avila S, McDaniel K, McMillin M, Afroze S, Guerrier M, Quezada M, Ray D, Kennedy L, Hargrove L (2014). Overexpression of membrane metalloendopeptidase inhibits substance P stimulation of cholangiocarcinoma growth. American journal of physiology Gastrointestinal and liver physiology.

[29] Seo PS, Quinn BJ, Khan AA, Zeng L, Takoudis CG, Hanada T, Bolis A, Bolino A, Chishti AH (2009). Identification of erythrocyte p55/MPP1 as a binding partner of NF2 tumor suppressor protein/Merlin. Exp Biol Med (Maywood).

[30] Kibel A, Iliopoulos O, DeCaprio JA, Kaelin WG (1995). Binding of the von Hippel-Lindau tumor suppressor protein to Elongin B and C. Science.

[31] Soldatenkov VA, Trofimova IN, Rouzaut A, McDermott F, Dritschilo A, Notario V (2002). Differential regulation of the response to DNA damage in Ewing's sarcoma cells by ETS1 and EWS/FLI-1. Oncogene.

[32] Melo FR, Grujic M, Spirkoski J, Calounova G, Pejler G (2012). Serglycin proteoglycan promotes apoptotic versus necrotic cell death in mast cells. The Journal of biological chemistry.

[33] Ma B, Zhong L, van Blitterswijk CA, Post JN, Karperien M (2013). T cell factor 4 is a pro-catabolic and apoptotic factor in human articular chondrocytes by potentiating nuclear factor kappaB signaling. The Journal of biological chemistry.

[34] Li X, Lu D, He F, Zhou H, Liu Q, Wang Y, Shao C, Gong Y (2011). Cullin 4B protein ubiquitin ligase targets peroxiredoxin III for degradation. The Journal of biological chemistry.

[35] Malina A, Cencic R, Pelletier J (2011). Targeting translation dependence in cancer. Oncotarget.

[36] Burch TC, Watson MT, Nyalwidhe JO (2013). Variable metastatic potentials correlate with differential plectin and vimentin expression in syngeneic androgen independent prostate cancer cells. PloS one.

[37] Niwa T, Saito H, Imajoh-ohmi S, Kaminishi M, Seto Y, Miki Y, Nakanishi A (2009). BRCA2 interacts with the cytoskeletal linker protein plectin to form a complex controlling centrosome localization. Cancer science.

[38] Parmigiani RB, Xu WS, Venta-Perez G, Erdjument-Bromage H, Yaneva M, Tempst P, Marks PA (2008). HDAC6 is a specific deacetylase of peroxiredoxins and is involved in redox regulation. Proceedings of the National Academy of Sciences of the United States of America.

[39] Wei TT, Lin YC, Lin PH, Shih JY, Chou CW, Huang WJ, Yang YC, Hsiao PW, Chen CC (2015). Induction of c-Cbl contributes to anti-cancer effects of HDAC inhibitor in lung cancer. Oncotarget.

[40] Wendt MK, Johanesen PA, Kang-Decker N, Binion DG, Shah V, Dwinell MB (2006). Silencing of epithelial CXCL12 expression by DNA hypermethylation promotes colonic carcinoma metastasis. Oncogene.

[41] Ganghammer S, Hutterer E, Hinterseer E, Brachtl G, Asslaber D, Krenn PW, Girbl T, Berghammer P, Geisberger R, Egle A, Zucchetto A, Kruschinski A, Gattei V, Chigaev A, Greil R, Hartmann TN (2015). CXCL12-induced VLA-4 activation is impaired in trisomy 12 chronic lymphocytic leukemia cells: a role for CCL21. Oncotarget.

[42] Broderick SR, Golas BJ, Pham D, Towe CW, Talbot SG, Kaufman A, Bains S, Huryn LA, Yonekawa Y, Carlson D, Hambardzumyan D, Ramanathan Y, Singh B (2010). SCCRO promotes glioma formation and malignant progression in mice. Neoplasia.

[43] Sarkaria IS, Stojadinovic A, Talbot SG, Hoos A, Dudas ME, Brennan MF, Ghossein RA, Singh B (2004). Squamous cell carcinoma-related oncogene is highly expressed in developing, normal, and adenomatous adrenal tissue but not in aggressive adrenocortical carcinomas. Surgery.

[44] Wang C, Jin G, Jin H, Wang N, Luo Q, Zhang Y, Gao D, Jiang K, Gu D, Shen Q, Huo X, Hu F, Ge T, Zhao F, Chu W, Shu H (2015). Clusterin facilitates metastasis by EIF3I/Akt/MMP13 signaling in hepatocellular carcinoma. Oncotarget.

[45] Kaspar M, Zardi L, Neri D (2006). Fibronectin as target for tumor therapy. International journal of cancer Journal international du cancer.

[46] Rosano L, Spinella F, Bagnato A (2013). Endothelin 1 in cancer: biological implications and therapeutic opportunities. Nature reviews Cancer.

[47] Pavet V, Shlyakhtina Y, He T, Ceschin DG, Kohonen P, Perala M, Kallioniemi O, Gronemeyer H (2014). Plasminogen activator urokinase expression reveals TRAIL responsiveness and supports fractional survival of cancer cells. Cell death & disease.

[48] Zhang GY, Ahmed N, Riley C, Oliva K, Barker G, Quinn MA, Rice GE (2005). Enhanced expression of peroxisome proliferator-activated receptor gamma in epithelial ovarian carcinoma. British journal of cancer.

[49] Wu N, Liu S, Guo C, Hou Z, Sun MZ (2013). The role of annexin A3 playing in cancers. Clinical & translational oncology: official publication of the Federation of Spanish Oncology Societies and of the National Cancer Institute of Mexico.

[50] Cao L, Shao M, Schilder J, Guise T, Mohammad KS, Matei D (2012). Tissue transglutaminase links TGF-beta, epithelial to mesenchymal transition and a stem cell phenotype in ovarian cancer. Oncogene.

[51] Eckhoff K, Flurschutz R, Trillsch F, Mahner S, Janicke F, Milde-Langosch K (2013). The prognostic significance of Jun transcription factors in ovarian cancer. Journal of cancer research and clinical oncology.

[52] Gilmour LM, Macleod KG, McCaig A, Sewell JM, Gullick WJ, Smyth JF, Langdon SP (2002). Neuregulin expression, function, and signaling in human ovarian cancer cells. Clinical cancer research: an official journal of the American Association for Cancer Research.

[53] Poli Neto OB, Candido Dos Reis FJ, Zambelli Ramalho LN, Nogueira AA, de Andrade JM (2006). p63 expression in epithelial ovarian tumors. International journal of gynecological cancer : official journal of the International Gynecological Cancer Society.

[54] Mandelin E, Lassus H, Seppala M, Leminen A, Gustafsson JA, Cheng G, Butzow R, Koistinen R (2003). Glycodelin in ovarian serous carcinoma: association with differentiation and survival. Cancer research.

[55] Hussein-Fikret S, Fuller PJ (2005). Expression of nuclear receptor coregulators in ovarian stromal and epithelial tumours. Molecular and cellular endocrinology.

[56] Philp AJ, Campbell IG, Leet C, Vincan E, Rockman SP, Whitehead RH, Thomas RJ, Phillips WA (2001). The phosphatidylinositol 3′-kinase p85alpha gene is an oncogene in human ovarian and colon tumors. Cancer research.

[57] Dong Z, Yang L, Lai D (2013). KLF5 strengthens drug resistance of ovarian cancer stem-like cells by regulating survivin expression. Cell proliferation.

[58] Kolligs FT, Nieman MT, Winer I, Hu G, Van Mater D, Feng Y, Smith IM, Wu R, Zhai Y, Cho KR, Fearon ER (2002). ITF-2, a downstream target of the Wnt/TCF pathway, is activated in human cancers with beta-catenin defects and promotes neoplastic transformation. Cancer cell.

[59] Huang YH, Bao Y, Peng W, Goldberg M, Love K, Bumcrot DA, Cole G, Langer R, Anderson DG, Sawicki JA (2009). Claudin-3 gene silencing with siRNA suppresses ovarian tumor growth and metastasis. Proceedings of the National Academy of Sciences of the United States of America.

[60] Spizzo G, Went P, Dirnhofer S, Obrist P, Moch H, Baeuerle PA, Mueller-Holzner E, Marth C, Gastl G, Zeimet AG (2006). Overexpression of epithelial cell adhesion molecule (Ep-CAM) is an independent prognostic marker for reduced survival of patients with epithelial ovarian cancer. Gynecologic oncology.

[61] Rask K, Zhu Y, Wang W, Hedin L, Sundfeldt K (2006). Ovarian epithelial cancer: a role for PGE2-synthesis and signalling in malignant transformation and progression. Molecular cancer.

[62] Pujol P, Rey JM, Nirde P, Roger P, Gastaldi M, Laffargue F, Rochefort H, Maudelonde T (1998). Differential expression of estrogen receptor-alpha and -beta messenger RNAs as a potential marker of ovarian carcinogenesis. Cancer research.

[63] Chen Q, Xu R, Zeng C, Lu Q, Huang D, Shi C, Zhang W, Deng L, Yan R, Rao H, Gao G, Luo S (2014). Down-regulation of Gli transcription factor leads to the inhibition of migration and invasion of ovarian cancer cells via integrin beta4-mediated FAK signaling. PloS one.

[64] Laviolette LA, Hodgkinson KM, Minhas N, Perez-Iratxeta C, Vanderhyden BC (2014). 17beta-estradiol upregulates GREB1 and accelerates ovarian tumor progression *in vivo*. International journal of cancer Journal international du cancer.

[65] Hudson LG, Moss NM, Stack MS (2009). EGF-receptor regulation of matrix metalloproteinases in epithelial ovarian carcinoma. Future Oncol.

[66] Huang GS, Brouwer-Visser J, Ramirez MJ, Kim CH, Hebert TM, Lin J, Arias-Pulido H, Qualls CR, Prossnitz ER, Goldberg GL, Smith HO, Horwitz SB (2010). Insulin-like growth factor 2 expression modulates Taxol resistance and is a candidate biomarker for reduced disease-free survival in ovarian cancer. Clinical cancer research: an official journal of the American Association for Cancer Research.

[67] De Cecco L, Marchionni L, Gariboldi M, Reid JF, Lagonigro MS, Caramuta S, Ferrario C, Bussani E, Mezzanzanica D, Turatti F, Delia D, Daidone MG, Oggionni M, Bertuletti N, Ditto A, Raspagliesi F (2004). Gene expression profiling of advanced ovarian cancer: characterization of a molecular signature involving fibroblast growth factor 2. Oncogene.

[68] Inoue M, Ogawa H, Miyata M, Shiozaki H, Tanizawa O (1992). Expression of E-cadherin in normal, benign, and malignant tissues of female genital organs. American journal of clinical pathology.

[69] Wilson AJ, Fadare O, Beeghly-Fadiel A, Son DS, Liu Q, Zhao S, Saskowski J, Uddin MJ, Daniel C, Crews B, Lehmann BD, Pietenpol JA, Crispens MA, Marnett LJ, Khabele D (2015). Aberrant over-expression of COX-1 intersects multiple pro-tumorigenic pathways in high-grade serous ovarian cancer. Oncotarget.

[70] Kohsaka S, Hinohara K, Wang L, Nishimura T, Urushido M, Yachi K, Tsuda M, Tanino M, Kimura T, Nishihara H, Gotoh N, Tanaka S (2014). Epiregulin enhances tumorigenicity by activating the ERK/MAPK pathway in glioblastoma. Neuro-oncology.

[71] Kondoh N, Shuda M, Tanaka K, Wakatsuki T, Hada A, Yamamoto M (2001). Enhanced expression of S8, L12, L23a, L27 and L30 ribosomal protein mRNAs in human hepatocellular carcinoma. Anticancer research.

[72] McCormack MP, Young LF, Vasudevan S, de Graaf CA, Codrington R, Rabbitts TH, Jane SM, Curtis DJ (2010). The Lmo2 oncogene initiates leukemia in mice by inducing thymocyte self-renewal. Science.

[73] Lee CC, Chen WS, Chen CC, Chen LL, Lin YS, Fan CS, Huang TS (2012). TCF12 protein functions as transcriptional repressor of E-cadherin, and its overexpression is correlated with metastasis of colorectal cancer. The Journal of biological chemistry.

[74] Favaro P, Traina F, Machado-Neto JA, Lazarini M, Lopes MR, Pereira JK, Costa FF, Infante E, Ridley AJ, Saad ST (2013). FMNL1 promotes proliferation and migration of leukemia cells. Journal of leukocyte biology.

[75] Lan L, Han H, Zuo H, Chen Z, Du Y, Zhao W, Gu J, Zhang Z (2010). Upregulation of myosin Va by Snail is involved in cancer cell migration and metastasis. International journal of cancer Journal international du cancer.

[76] Huang HL, Chiang CH, Hung WC, Hou MF (2015). Targeting of TGF-beta-activated protein kinase 1 inhibits chemokine (C-C motif) receptor 7 expression, tumor growth and metastasis in breast cancer. Oncotarget.

[77] Li H, Mohamed AA, Sharad S, Umeda E, Song Y, Young D, Petrovics G, McLeod DG, Sesterhenn IA, Sreenath T, Dobi A, Srivastava S (2015). Silencing of PMEPA1 accelerates the growth of prostate cancer cells through AR, NEDD4 and PTEN. Oncotarget.

[78] Guo C, Liu S, Sun MZ (2013). Potential role of Anxa1 in cancer. Future Oncol.

[79] Olkhanud PB, Rochman Y, Bodogai M, Malchinkhuu E, Wejksza K, Xu M, Gress RE, Hesdorffer C, Leonard WJ, Biragyn A (2011). Thymic stromal lymphopoietin is a key mediator of breast cancer progression. J Immunol.

[80] Deblois G, Hall JA, Perry MC, Laganiere J, Ghahremani M, Park M, Hallett M, Giguere V (2009). Genome-wide identification of direct target genes implicates estrogen-related receptor alpha as a determinant of breast cancer heterogeneity. Cancer research.

[81] Yasumaru M, Tsuji S, Tsujii M, Irie T, Komori M, Kimura A, Nishida T, Kakiuchi Y, Kawai N, Murata H, Horimoto M, Sasaki Y, Hayashi N, Kawano S, Hori M (2003). Inhibition of angiotensin II activity enhanced the antitumor effect of cyclooxygenase-2 inhibitors via insulin-like growth factor I receptor pathway. Cancer research.

[82] Drayton RM, Peter S, Myers K, Miah S, Dudziec E, Bryant HE, Catto JW (2014). MicroRNA-99a and 100 mediated upregulation of FOXA1 in bladder cancer. Oncotarget.

[83] Murthy SR, Pacak K, Loh YP (2010). Carboxypeptidase E: elevated expression correlated with tumor growth and metastasis in pheochromocytomas and other cancers. Cellular and molecular neurobiology.

[84] Bee A, Ke Y, Forootan S, Lin K, Beesley C, Forrest SE, Foster CS (2006). Ribosomal protein l19 is a prognostic marker for human prostate cancer. Clinical cancer research : an official journal of the American Association for Cancer Research.

[85] Nimmagadda D, Cherala G, Ghatta S (2006). Cytosolic sulfotransferases. Indian journal of experimental biology.

[86] Brozic P, Turk S, Rizner TL, Gobec S (2011). Inhibitors of aldo-keto reductases AKR1C1-AKR1C4. Current medicinal chemistry.

[87] Pallai R, Simpkins H, Chen J, Parekh HK (2010). The CCAAT box binding transcription factor, nuclear factor-Y (NF-Y) regulates transcription of human aldo-keto reductase 1C1 (AKR1C1) gene. Gene.

[88] Kim S, You S, Hwang D (2011). Aminoacyl-tRNA synthetases and tumorigenesis: more than housekeeping. Nature reviews Cancer.

[89] Xu X, Zhao J, Xu Z, Peng B, Huang Q, Arnold E, Ding J (2004). Structures of human cytosolic NADP-dependent isocitrate dehydrogenase reveal a novel self-regulatory mechanism of activity. The Journal of biological chemistry.

[90] Bleeker FE, Lamba S, Leenstra S, Troost D, Hulsebos T, Vandertop WP, Frattini M, Molinari F, Knowles M, Cerrato A, Rodolfo M, Scarpa A, Felicioni L, Buttitta F, Malatesta S, Marchetti A (2009). IDH1 mutations at residue p.R132 (IDH1(R132)) occur frequently in high-grade gliomas but not in other solid tumors. Human mutation.

[91] Petersen AK, Stark K, Musameh MD, Nelson CP, Romisch-Margl W, Kremer W, Raffler J, Krug S, Skurk T, Rist MJ, Daniel H, Hauner H, Adamski J, Tomaszewski M, Doring A, Peters A (2012). Genetic associations with lipoprotein subfractions provide information on their biological nature. Human molecular genetics.

[92] DeBerardinis RJ (2011). Serine metabolism: some tumors take the road less traveled. Cell metabolism.

[93] Chen J, Chung F, Yang G, Pu M, Gao H, Jiang W, Yin H, Capka V, Kasibhatla S, Laffitte B, Jaeger S, Pagliarini R, Chen Y, Zhou W (2013). Phosphoglycerate dehydrogenase is dispensable for breast tumor maintenance and growth. Oncotarget.

[94] Amadori D, Sansoni E, Amadori A (1997). Ovarian cancer: natural history and metastatic pattern. Frontiers in bioscience: a journal and virtual library.

[95] Feki A, Berardi P, Bellingan G, Major A, Krause KH, Petignat P, Zehra R, Pervaiz S, Irminger-Finger I (2009). Dissemination of intraperitoneal ovarian cancer: Discussion of mechanisms and demonstration of lymphatic spreading in ovarian cancer model. Critical reviews in oncology/hematology.

[96] Garson K, Shaw TJ, Clark KV, Yao DS, Vanderhyden BC (2005). Models of ovarian cancer—are we there yet?. Molecular and cellular endocrinology.

[97] Taylor PT, Haverstick D (2005). Re: New guidelines to evaluate the response to treatment in solid tumors (ovarian cancer). Journal of the National Cancer Institute.

[98] Keita M, Bachvarova M, Morin C, Plante M, Gregoire J, Renaud MC, Sebastianelli A, Trinh XB, Bachvarov D (2013). The RUNX1 transcription factor is expressed in serous epithelial ovarian carcinoma and contributes to cell proliferation, migration and invasion. Cell cycle.

[99] Tetu B, Popa I, Bairati I, L'Esperance S, Bachvarova M, Plante M, Harel F, Bachvarov D (2008). Immunohistochemical analysis of possible chemoresistance markers identified by micro-arrays on serous ovarian carcinomas. Modern pathology : an official journal of the United States and Canadian Academy of Pathology, Inc.

[100] Mercier PL, Bachvarova M, Plante M, Gregoire J, Renaud MC, Ghani K, Tetu B, Bairati I, Bachvarov D (2011). Characterization of DOK1, a candidate tumor suppressor gene, in epithelial ovarian cancer. Molecular oncology.

[101] Wang ZQ, Bachvarova M, Morin C, Plante M, Gregoire J, Renaud MC, Sebastianelli A, Bachvarov D (2014). Role of the polypeptide N-acetylgalactosaminyltransferase 3 in ovarian cancer progression: possible implications in abnormal mucin O-glycosylation. Oncotarget.

[102] Wang ZQ, Keita M, Bachvarova M, Gobeil S, Morin C, Plante M, Gregoire J, Renaud MC, Sebastianelli A, Trinh XB, Bachvarov D (2013). Inhibition of RUNX2 Transcriptional Activity Blocks the Proliferation, Migration and Invasion of Epithelial Ovarian Carcinoma Cells. PloS one.

[103] Römisch-Margl W, Prehn C, Bogumil R, Rohring C, Suhre K, Adamski J (2012). Procedure for tissue sample preparation and metabolite extraction for high-throughput targeted metabolomics. Metabolomics : Official journal of the Metabolomic Society.

