# Supplementary material for: BCAT1 expression associates with ovarian cancer progression: possible implications in altered disease metabolism
Source: Oncotarget. 2015 Sep 10;6(31):31522–43. doi: 10.18632/oncotarget.5159 (PMC4741622; doi:10.18632/oncotarget.5159)
Supplement: Supplementary file 1 [file oncotarget-06-31522-s001.pdf]

SUPPLEMENTARY FIGURES AND TABLES

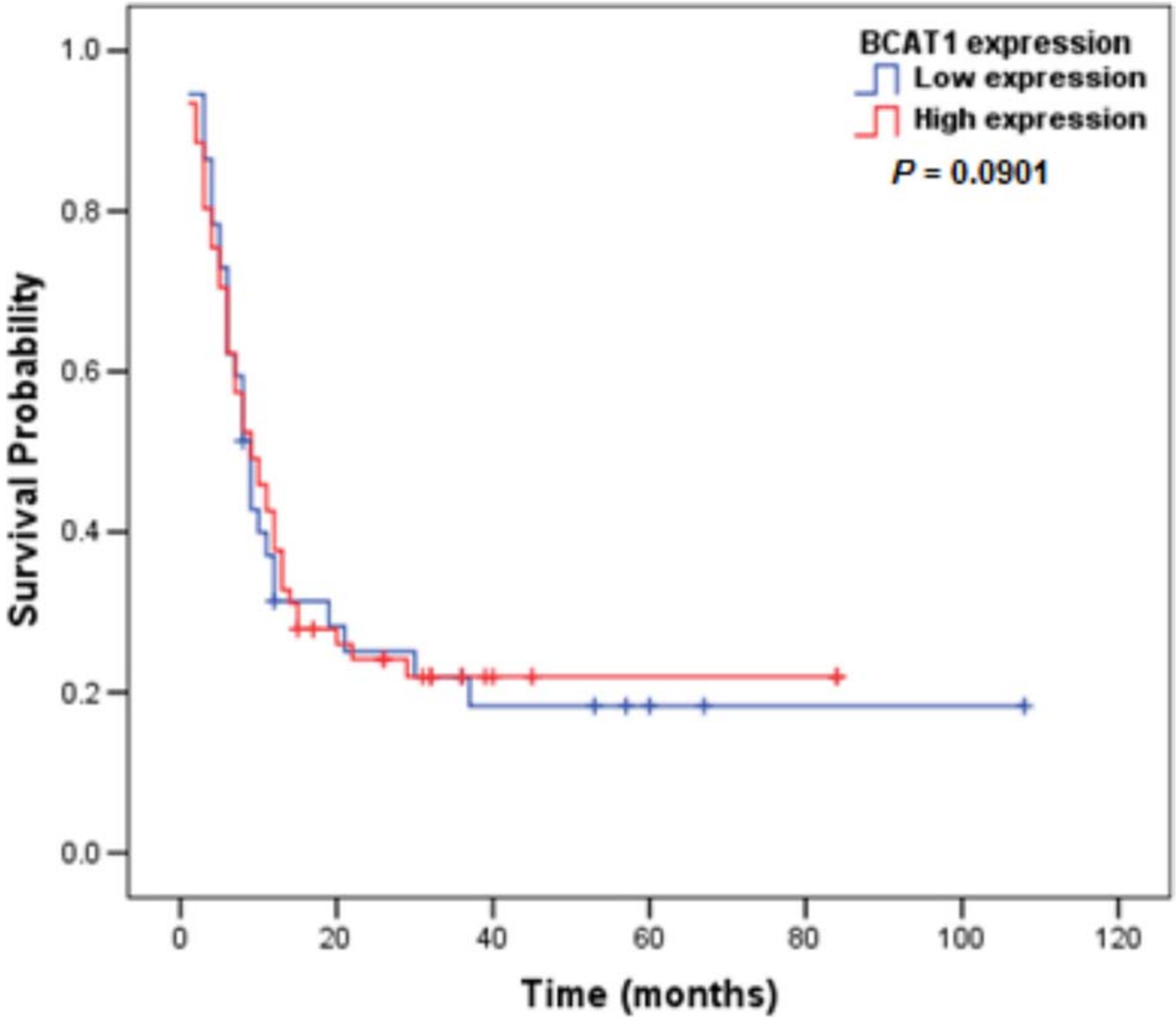

Supplementary Figure S1: Kaplan-Meier curve for progression free survival according to the level of BCAT1 IHC intensity in tumor samples of 103 serous EOC patients.

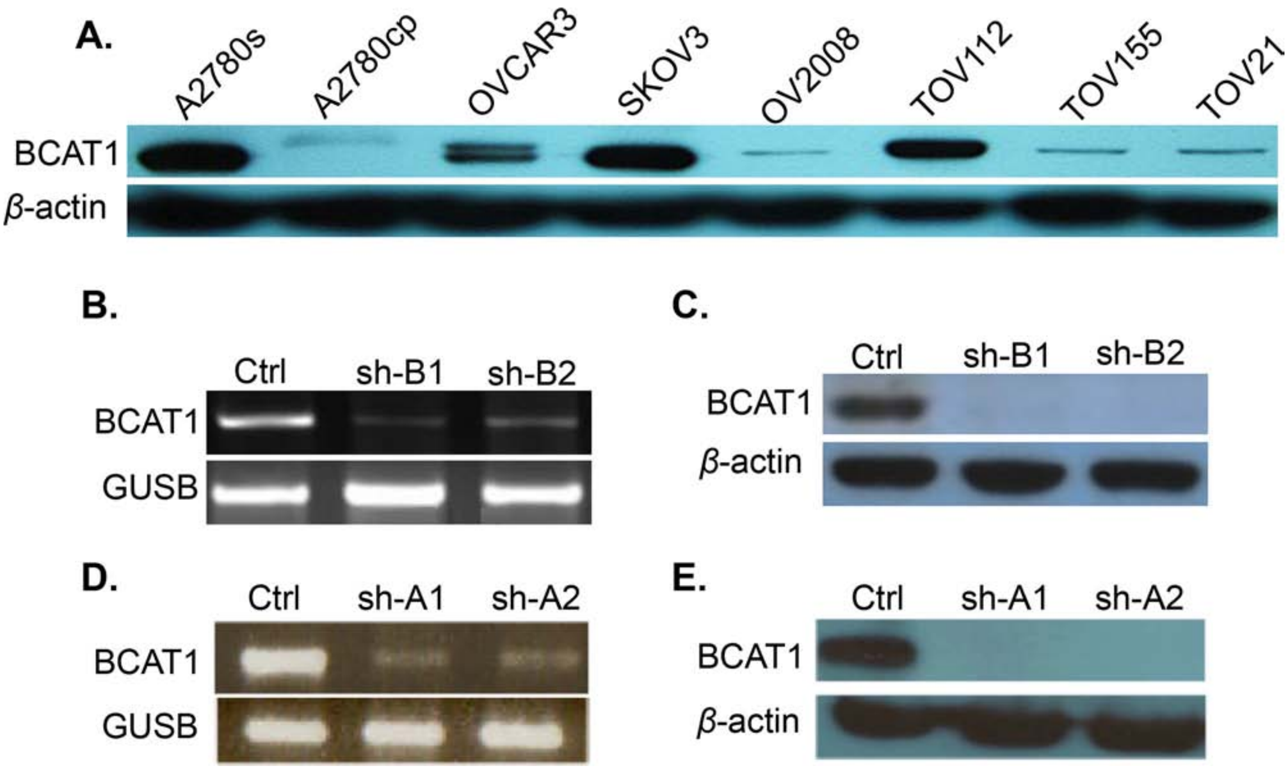

Supplementary Figure S2: Analysis of BCAT1 expression in EOC cell lines. A. Displayed are images of representative results following Western-blot analysis of BCAT1 protein expression in different EOC cell lines. **B.** Semi-quantitative duplex RT-PCR (sqRT-PCR) analysis of BCAT1 mRNA expression levels in the shRNA-BCAT1 knockdown SKOV3 cell clones 1 and 2 (sh-B1 and sh-B2), compared to the mock-transfected control SKOV3 clone (Ctrl). Displayed are images of representative results following sqRT-PCR analysis. The GUSB gene was used as internal standard. **C.** Western-blot analysis of BCAT1 protein expression in clones sh-B1 and sh-B2, compared to the Ctrl clone. *β*-actin was used as a loading control. D. sqRT-PCR analysis of BCAT1 mRNA expression levels in the shRNA-BCAT1 knockdown A2780s cell clones 1 and 2 (sh-A1 and sh-A2), compared to the mock-transfected control A2780s clone (Ctrl). D. Western-blot analysis of BCAT1 protein expression in clones sh-A1 and sh-A, compared to the control A2780s clone (Ctrl).

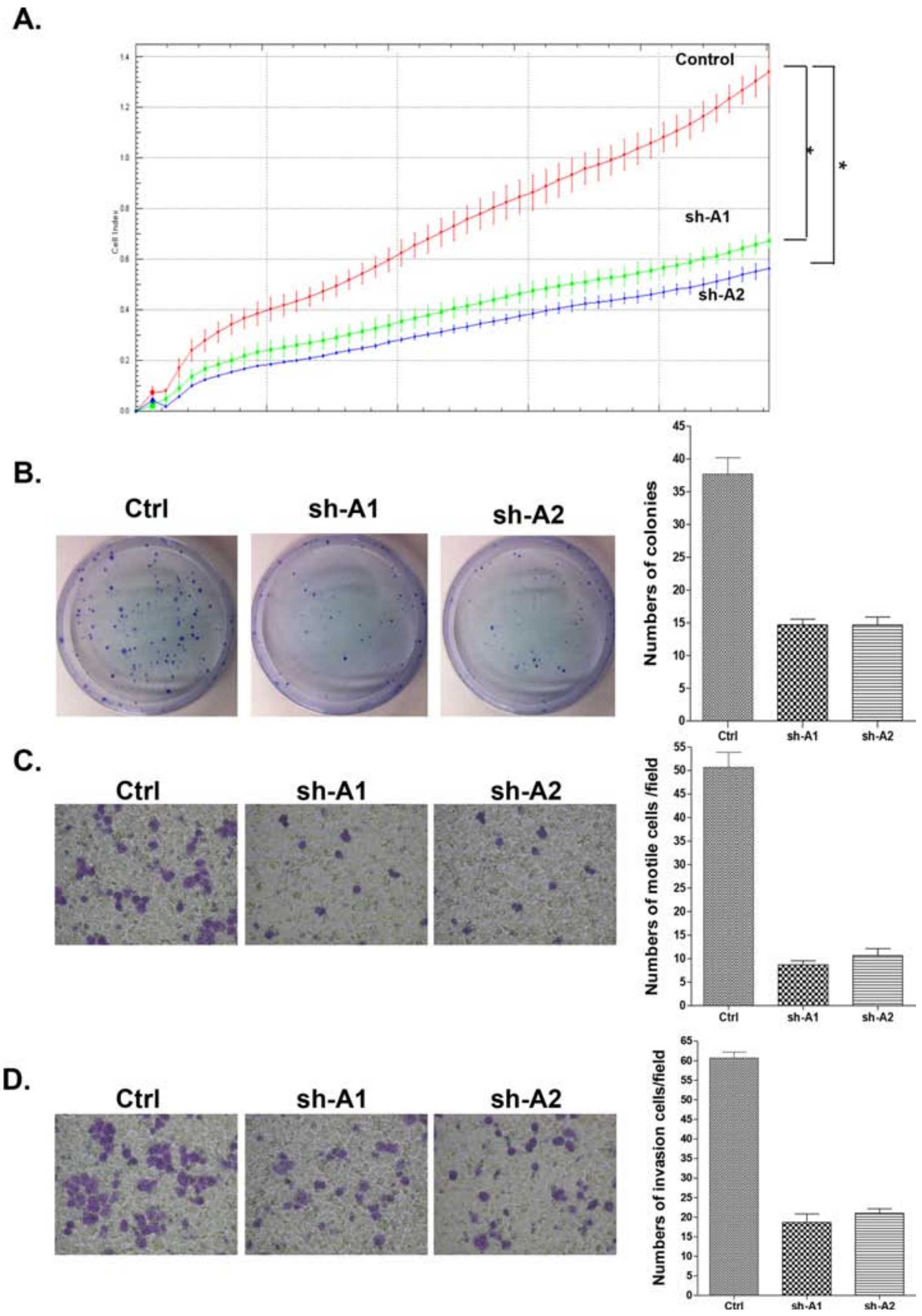

Supplementary Figure S3: ShRNA-mediated knockdown of the BCAT1 expression in A2780s cells and consecutive analyses of functional phenotypes. A. effect of BCAT1 knockdown clones sh-A1 and sh-A2 on cell proliferation, compared to the control clone (Ctrl). **B.** Representative images of colony formation assays following BCAT1 knockdown (left) and graph bars presentation of colony numbers in the Ctrl clone and shRNA-BCAT1 clones sh-A1, sh-A2 (right). D, E, Representative images from one of the three independent experiments showing migration (D, left) and invasion (E, left) in the control clone and clones sh-A1 and sh-A2 (at magnification × 400). The bar graphs in panels D (*right*) and E (*right*) are quantitative determinations of data obtained by selecting 10 random fields per filter (at magnification× 40) under phase contrast microscopy. Differences between shRNA-BCAT1-transfected and vehicle-transfected A2780s cells were determined by a Student's *t*-test. Error bars denote ± SEM and *indicates statistical significance ($P < 0.05$).

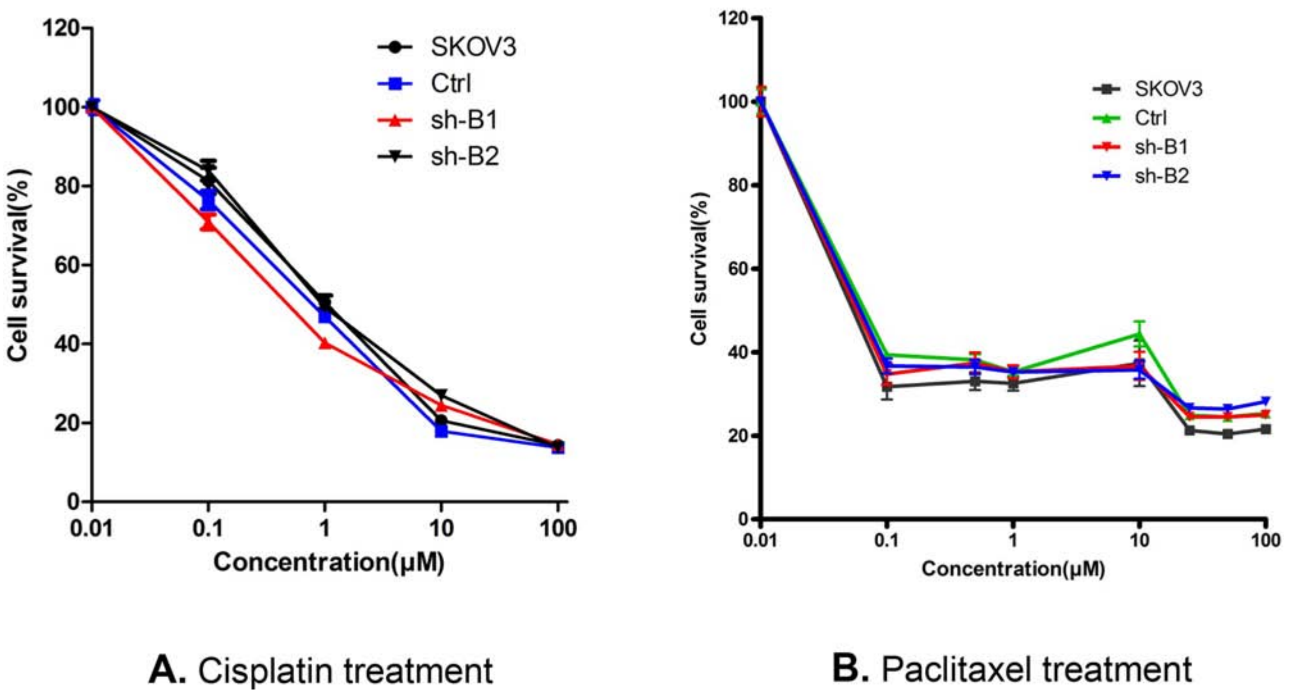

Supplementary Figure S4: Dose-response cytotoxicity curves upon cisplatin A. and paclitaxel **B.** treatment of SKOV3 cells following shRNA-mediated BCAT1 knockdown. Treatment responses of the shRNA-BCAT1 clones sh-B1 and sh-B2 were compared to the mock-transfected control (ctrl) clone following 72 hours incubation with the corresponding drug. Error bars denote ± SEM. All the *P* value are >0.05 according to the one-way ANOVA analysis.

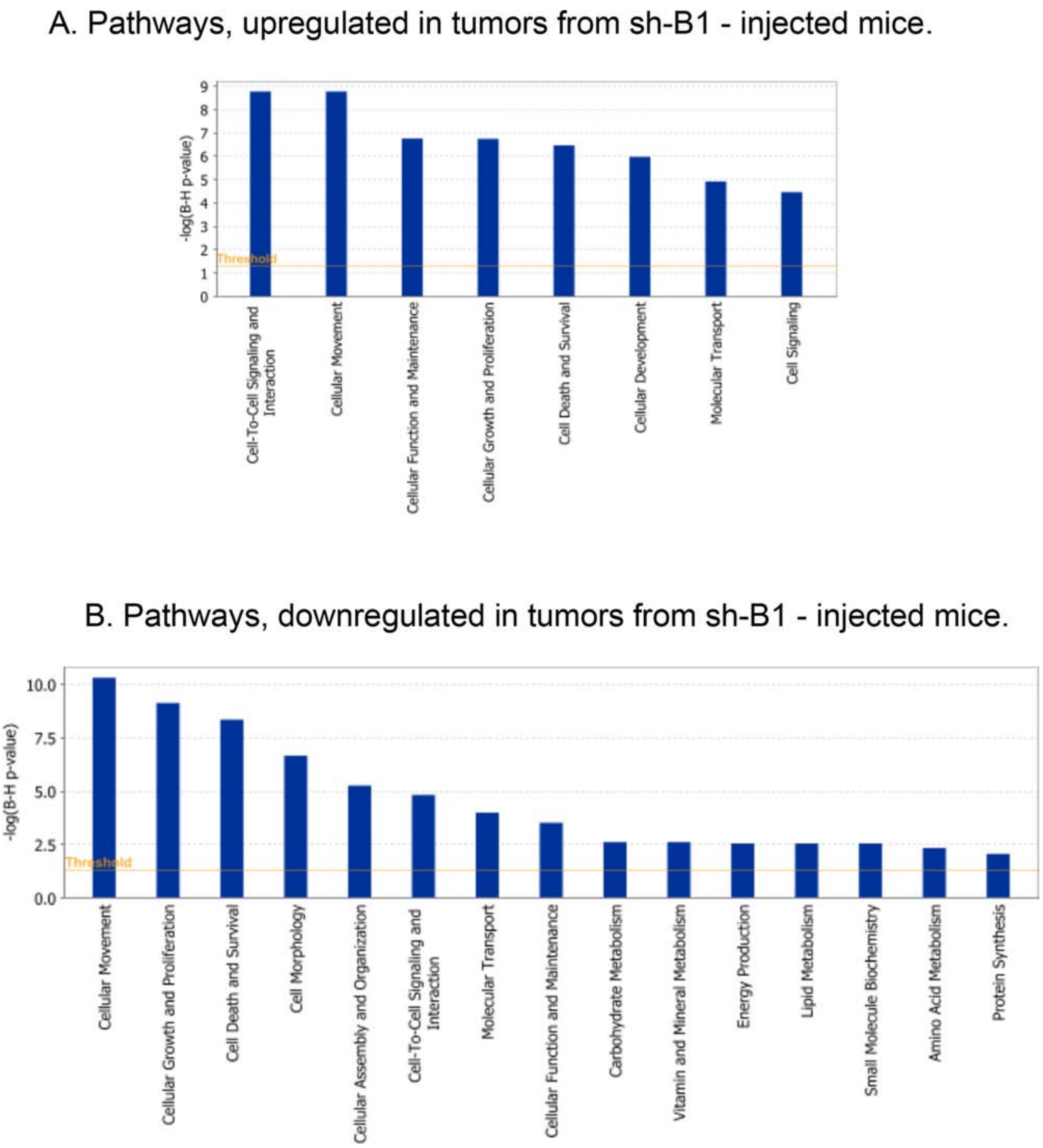

Supplementary Figure S5: Comparison for datasets of functionally-related differentially expressed genes (≥2-fold) in tumor tissues extracted from two nude mice IP injected with BCAT1 knockdown (sh-B1) cells versus tumors from two nude mice IP injected with mock-transfected (Ctrl) cells. The microarray experiments were performed in duplicates, as two hybridizations were carried out for each of the two tumors extracted from the sh-B1-injected mice against the corresponding controls (tumors from Ctrl-injected mice), using a fluorescent dye reversal (dye-swap) technique. Total RNA extraction from tumor samples and consecutive global gene expression analyses were performed as described in the Methods section. **A.** Functional analyses of upregulated genes, **B.** Functional analyses of downregulated genes. Top functions that meet a *p*-value cutoff of 0.05 are displayed.

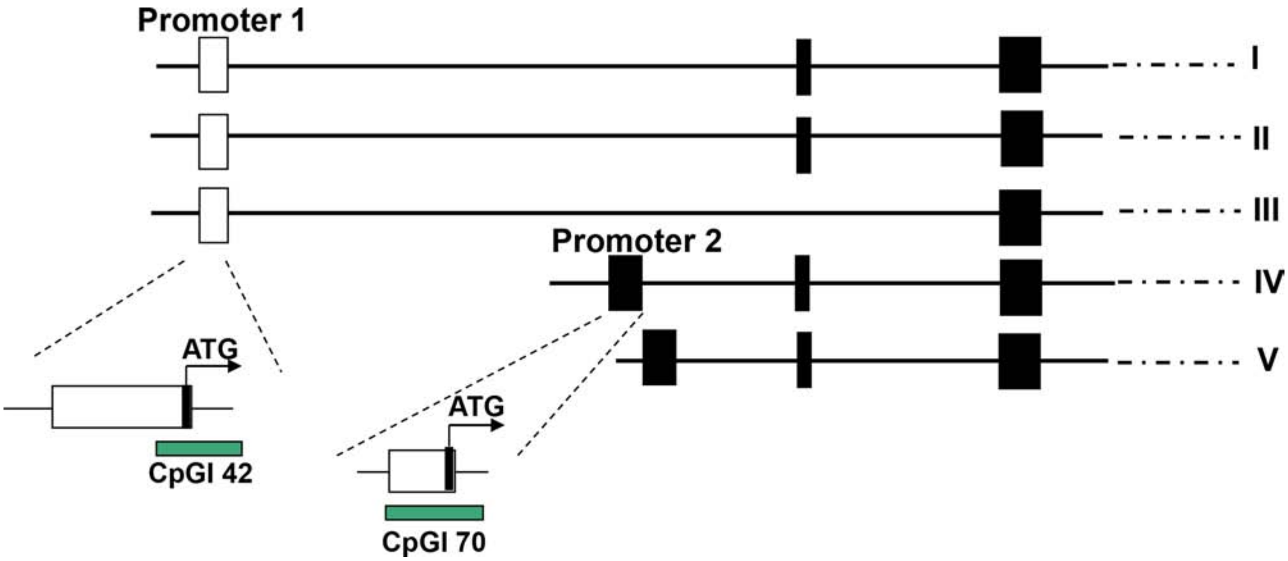

Supplementary Figure S6: Genomic structure of the BCAT1 gene, isoforms I, II, III, IV and V. The CpG island (CpGI) is indicated with arrow.

Supplementary Table S1: Genes, differentially expressed in SKOV3 cells (≥2.0 fold, *P* ≤ 0.05) following BCAT1 knockdown.

Supplementary Table S2: Primers for qPCR and sqRT-PCR.

Gene	Tm(°C)	Length(bp)	Forward	Reverse
**A. qPCR primers**				
TGFBI	60	126	CCAAGAGAACGGAGCAGACT	TTGGAGAGACTTTAGCCGCA
THBS1	60	136	GGGACGATGACTATGCTGGA	ACAGAAAGGCCCGAGTATCC
ZP1	60	134	AAATGGTAGCCTTGGACGGG	AGAGGTGCTGCAGAACAAGT
ADAMTS5	60	128	TTCTTGCAACAGACCCCACT	CTGCGAAGTGTGTGATCCCA
S100P	60	136	GGTGCTGATGGAGAAGGAGC	AGCCACGAACACGATGAACT
GREB1	60	138	TCACCCCTCACTAAACGCAG	GGCCCGGAGTTTGACAAGAT
RXFP1	60	149	CCCATTAACAGTGCTTTGAACCC	ACCCAGATGAATGATGGAGCA
GDF15	60	126	AGATCAAGACGAGCCTGCAC	CATAGGTCTGGAGCGACACC
IL1A	60	130	TGAAGACCAACCAGTGCTGC	TGGATGGGCAACTGATGTGA
SLPI	60	140	CCTGGATCCTGTTGACACCC	TGCAACACTTCAAGTCACGC
IGFBP2	60	149	ATCTCCACCATGCGCCTTC	TTCACACACCAGCACTCCC
18S	60	119	AACCCGTTGAACCCCATT	CCATCCAATCGGTAGTAGCG
**B. sqRT-PCR primers**				
BCAT1	60	157	AAGAACTGGCAACTCCTCCA	CTCACTCTGTTCCCCTCCAG
GUSB	60	187	ATACGTGGTTGGAGAGCTCATT	CTTGGCTACTGAGTGGGGATAC
